# Nociception and pain in humans lacking a functional TRPV1 channel

**DOI:** 10.1172/JCI153558

**Published:** 2023-02-01

**Authors:** Ben Katz, Rachel Zaguri, Simon Edvardson, Channa Maayan, Orly Elpeleg, Shaya Lev, Elyad Davidson, Maximilian Peters, Shlomit Kfir-Erenfeld, Esther Berger, Shifa Ghazalin, Alexander M. Binshtok, Baruch Minke

**Affiliations:** 1 Department of Medical Neurobiology, Faculty of Medicine and the Edmond and Lily Safra Center for Brain Sciences (ELSC), The Hebrew University, Jerusalem, Israel.; 2 Pediatric Neurology Unit, Pediatric Department, Hadassah University Hospital, Mount Scopus, Jerusalem, Israel.; 3 Department of Human Genetics,; 4 Pain Relief Unit, Department of Anesthesiology and Critical Care Medicine, and; 5 Department of Bone Marrow Transplantation and Cancer Immunology, Hadassah University Hospital, Ein Kerem, Jerusalem, Israel.; 6 Department of Pathology, E. Wolfson Medical Center, Holon, Israel.

**Keywords:** Neuroscience, Ion channels, Pain

## Abstract

**BACKGROUND:**

Chronic pain is a debilitating illness with currently limited therapy, in part due to difficulties in translating treatments derived from animal models to patients. The transient receptor potential vanilloid 1 (TRPV1) channel is associated with noxious heat detection and inflammatory pain, and reports of adverse effects in human trials have hindered extensive efforts in the clinical development of TRPV1 antagonists as novel pain relievers.

**METHODS:**

We examined 2 affected individuals (A1 and A2) carrying a homozygous missense mutation in TRPV1, rendering the channel nonfunctional. Biochemical and functional assays were used to analyze the mutant channel. To identify possible phenotypes of the affected individuals, we performed psychophysical and medical examinations.

**RESULTS:**

We demonstrated that diverse TRPV1 activators, acting at different sites of the channel protein, were unable to open the cloned mutant channel. This finding was not a consequence of impairment in the expression, cellular trafficking, or assembly of protein subunits. The affected individuals were insensitive to application of capsaicin to the mouth and skin and did not demonstrate aversive behavior toward capsaicin. Furthermore, quantitative sensory testing of A1 revealed an elevated heat-pain threshold but also, surprisingly, an elevated cold-pain threshold and extensive neurogenic inflammatory, flare, and pain responses following application of the TRPA1 channel activator mustard oil.

**CONCLUSION:**

Our study provides direct evidence in humans for pain-related functional changes linked to TRPV1, which is a prime target in the development of pain relievers.

**FUNDING:**

Supported by the Israel Science Foundation (368/19); Teva’s National Network of Excellence in Neuroscience grant (no. 0394886) and Teva’s National Network of Excellence in Neuroscience postdoctoral fellowship.

## Graphical abstract

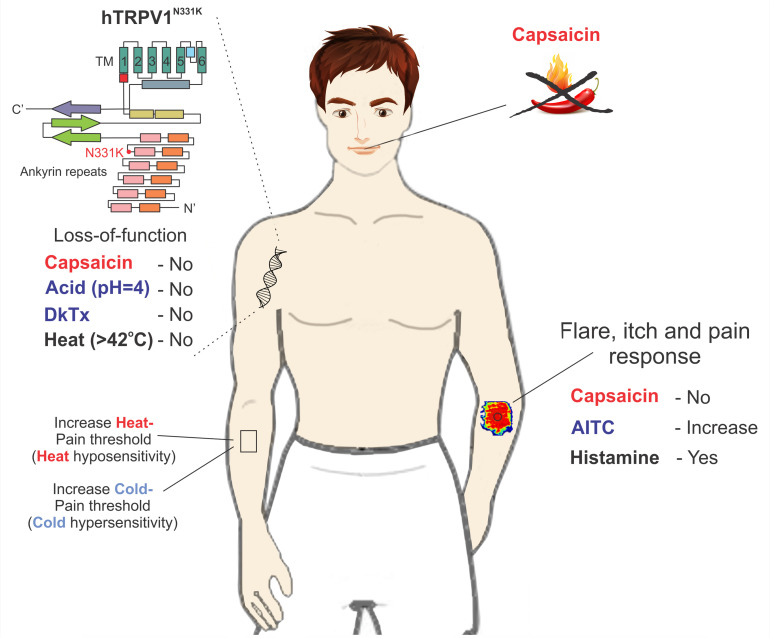

## Introduction

The ability to sense noxious stimuli is crucial for protecting organisms from tissue damage. The discovery of the capsaicin and heat-activated polymodal ion channel transient receptor potential vanilloid 1 (TRPV1) has advanced the molecular understanding of noxious heat sensation and inflammatory pain (1, 2). The TRPV1 channel is mainly expressed in somatic and visceral nociceptive neurons (3), but also in neurons of the CNS (4) and cells of the immune system (5). In rodents, the TRPV1 channel has been widely studied, and its activity is predominantly associated with detection of noxious heat (6) and the development of inflammatory hyperalgesia (7), whereas in humans, the functional roles of TRPV1 are not entirely clear and rely extensively on clinical pharmacological studies. These studies have revealed roles of TRPV1 in noxious heat sensation and thermoregulation (7, 8). A powerful methodology to obtain a better understanding of the functional roles of TRPV1 channels in humans (hTRPV1) is to identify individuals carrying deleterious mutations in the *TRPV1* gene. To our knowledge, only 2 studies that utilized the genetic approach have been reported for the *hTRPV1* gene. First is a study describing an individual carrying mutations in the second intron of *hTRPV1*, resulting in a reduction of approximately 65% of TRPV1 protein levels. These mutations caused insensitivity to capsaicin, hypersensitivity to garlic extract applied to the mouth, and similar, but slightly prolonged, response latencies to noxious heat compared with healthy volunteers (9). The second involves a study of patients with cystinosis, who harbored a 57 kb deletion in the *CTNS* gene, which also extends into the noncoding region upstream of the start codon of *hTRPV1*, leading to a large reduction of *TRPV1* mRNA expression levels (10). The homozygous patients harboring this deletion exhibited insensitivity to capsaicin, a slight (~2°C) increase in the warm detection threshold (WDT), and a heat-pain threshold (HPT) similar to that of healthy volunteers. Responses to cold and mechanical stimuli or cinnamaldehyde, an agonist of the TRPA1 channel, were unaltered (11). These studies emphasize the role of TRPV1 in capsaicin detection and show minor changes in noxious heat detection. However, given the complex genotype and its impact on TRPV1 expression in these studies, the conclusions regarding the functional roles of hTRPV1 are circumstantial. Here, we characterized and performed comprehensive medical and functional examinations of an individual carrying a yet-unidentified homozygous missense mutation in the *hTRPV1* gene that resulted in a total loss of function of the TRPV1 channel, providing a unique opportunity to study the functional role of this channel in humans.

## Results

**Figure 1:**
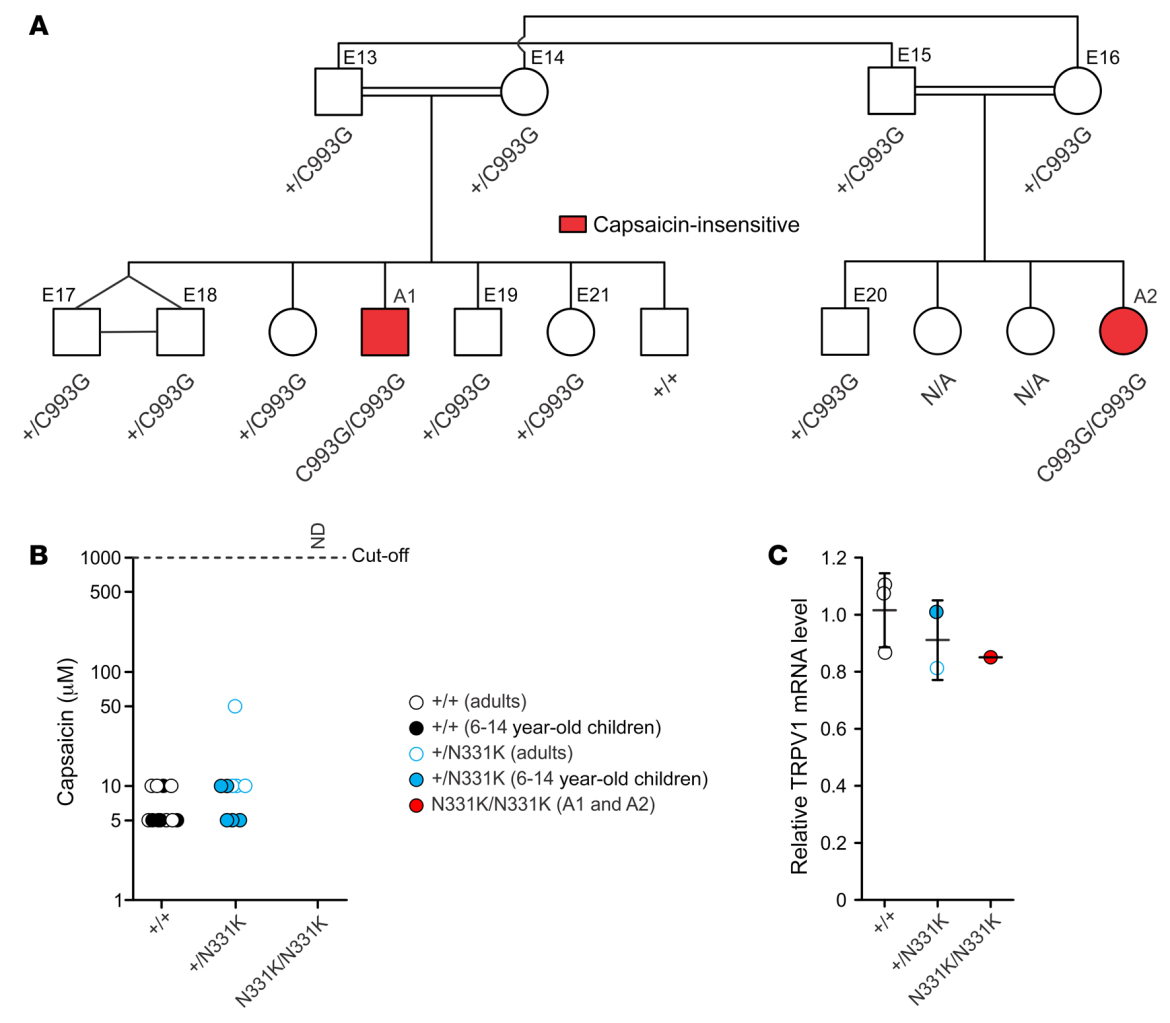
Genetic and molecular analysis of the affected individuals who are insensitive to capsaicin. (**A**) Pedigree chart of the family carrying a missense mutation of the *TRPV1* gene. Two affected individuals (A1 and A2) are the only homozygous family members carrying the c.993C>G mutation in the examined family; they are also the only family members who are insensitive to capsaicin (red), as shown in **B**. (**B**) Recognition threshold to capsaicin applied to the mouth. Capsaicin-sensitive participants responded to the compound as “hot” or “spicy” (healthy volunteers, *n*_+/+_ = 13; heterozygous family members, *n*_+/N331K_ = 9). Capsaicin-insensitive participants were identified as lacking any aversive behavior toward the application of capsaicin or responded to the compound applied as “water” (homozygous affected individuals, *n*_N331K/N331K_ = 2). The cutoff concentration of capsaicin was 1 mM, which was neither detected by A1 or A2 nor reported to cause any aversion (ND, not detected). (**C**) Relative *TRPV1* mRNA levels obtained from PBMCs of healthy volunteers (*n*_+/+_ = 3), heterozygous family members (*n*_+/N331K_ = 2), and A1 (mean of 4 repeats, see Supplemental Figure 1D) were determined by quantitative real-time PCR (error bars indicate the SD).

In this study, we examined a Palestinian Arab family, in which 1 of their children (affected individual 1 [A1], an 11-year-old male) eats large amounts of “hot” peppers without showing any signs of taste aversion, blotching, or tears when examined repeatedly from 3 years of age (Figure 1A). Sequencing of the *TRPV1* gene in A1 identified a homozygous missense mutation (c.993C>G) in exon 5 (Supplemental Figure 1A; supplemental material available online with this article; https://doi.org/10.1172/JCI153558DS1), which results in the substitution of an asparagine 331 by a lysine (N331K) located at finger 5 of the ankyrin repeat domain (ARD) of the channel (Supplemental Figure 1, B and C) (12, 13). The N331 residue is conserved in TRPV1 through evolution (Supplemental Figure 9A) and in all members of the hTRPV and hTRPC subfamilies (Supplemental Figure 1B), raising the possibility of a critical function of this site. We therefore performed further examinations of the proband (A1). Genotyping other members of the family along with a psychophysical test that examined capsaicin sensitivity of the mouth (Figure 1, A and B) revealed an additional affected individual (A2), a 1-year-old female, first-degree cousin of the proband, who carries the same homozygous N331K mutation and has no sensitivity to capsaicin, even at the high concentration of 1 mM (Figure 1B). In contrast, heterozygous family members showed normal capsaicin sensitivity compared with healthy volunteers (Figure 1B). Apart from insensitivity to capsaicin, A1 showed normal taste perception of a variety of tastants (Supplemental Figure 1E and Supplemental Table 2). Importantly, we measured similar levels of *TRPV1* mRNA in PBMCs from A1, his family members, and healthy volunteers (Figure 1C and Supplemental Figure 1D). This result infers that the expression levels of *TRPV1* in other tissues, and particularly in sensory neurons, may also be similar in A1 and the control groups. In conclusion, the results suggest that the N331K mutation has a strong effect on TRPV1 function without affecting its expression level.

### The hTRPV1^N331K^ channel does not respond to the common TRPV1 activators.

**Figure 2:**
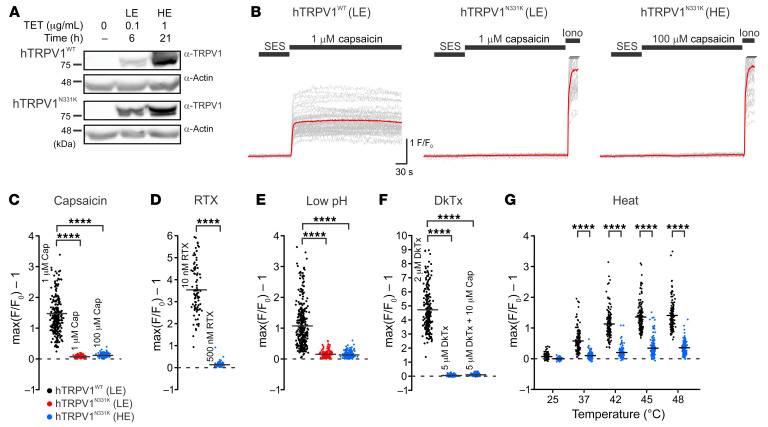
Functional analysis of the TRPV1^N331K^ channel. (**A**) Western blot analysis of T-REx-293 cells stably expressing hTRPV1^WT^ (clone 2) or hTRPV1^N331K^ (clone 2) at 2 different doses and durations of TET incubation, using anti-TRPV1 (α-TRPV1) antibody. α-Actin antibody was used as a protein loading control (*n* = 3). (**B**) Traces of Fura-2–based Ca^2+^ imaging from a representative plate of T-REx-293 cell lines stably expressing hTRPV1^WT^ at LE levels (left trace), hTRPV1^N331K^ at LE levels (middle trace), and hTRPV1^N331K^ at HE levels (right trace) in response to application of capsaicin (left and middle, 1 μM; right, 100 μM). Application of the Ca^2+^ ionophore ionomycin (Iono) served as a positive control (middle and right traces). The thick red line represents the mean trace. SES, standard extracellular solution. (**C**–**F**) Maximal (max) normalized fluorescence changes from baseline (see Methods) during the application of various TRPV1 agonists. (**C**) hTRPV1^WT^ LE, 1 μM capsaicin (Cap) (*N* = 5 plates, *n* = 287 cells), hTRPV1^N331K^ LE, 1 μM capsaicin (*N* = 3, *n* = 189), hTRPV1^N331K^ HE, 100 μM capsaicin (*N* = 3, *n* = 178); (**D**) hTRPV1^WT^ LE, 10 nM RTX (*N* = 2, *n* = 88), hTRPV1^N331K^ HE, 500 nM RTX (*N* = 2, *n* = 100); (**E**) hTRPV1^WT^ LE, solution of pH = 4 (low pH, *N* = 6, *n* = 340), hTRPV1^N331K^ LE, low pH (*N* = 2, *n* = 161), hTRPV1^N331K^ HE, low pH (*N* = 4, *n* = 232); (**F**) hTRPV1^WT^ LE, 2 μM DkTx (*N* = 2, *n* = 202), hTRPV1^N331K^ HE, 5 μM DkTx (*N* = 2, *n* = 204), hTRPV1^N331K^ HE, 5 μM DkTx plus 10 μM capsaicin (*N* = 2, *n* = 204). (**G**) Maximal normalized fluorescence changes from baseline during heat ramp from 25°C to 50°C at various temperatures: hTRPV1^WT^ LE, heat (*N* = 4, *n* = 155); hTRPV1^N331K^ HE, heat (*N* = 6, *n* = 155). ****P < 0.0001, by 2-tailed Student’s *t* test with Bonferroni’s correction for multiple comparisons (**C**–**F**) and 2-way ANOVA with Bonferroni’s correction for multiple comparisons (**G**).

**Figure 3:**
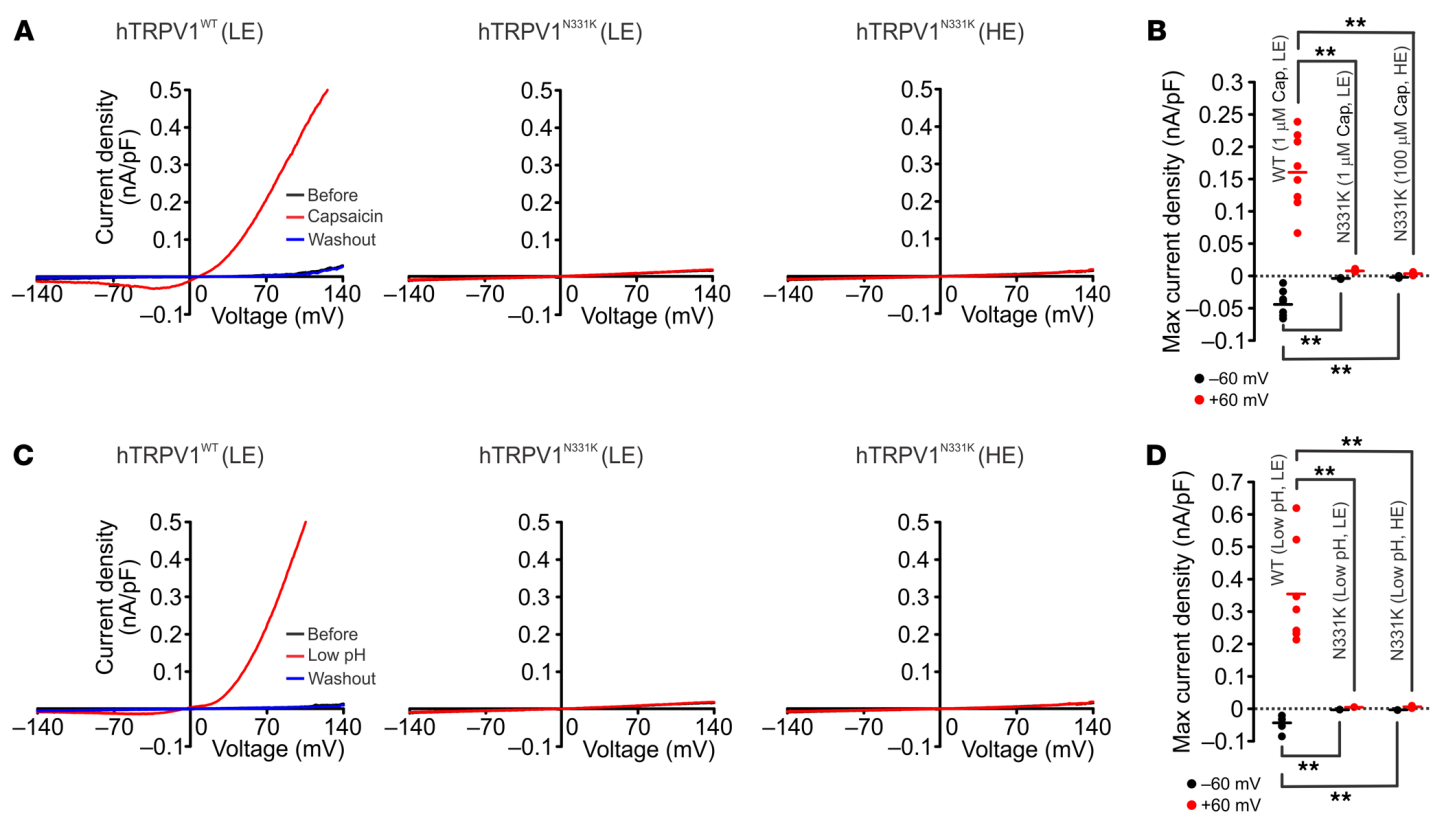
Electrophysiological analysis of the TRPV1^N331K^ channel. (**A**) Patch clamp whole-cell current measurements of T-REx-293 cell lines stably expressing hTRPV1^WT^ (left, LE) or hTRPV1^N331K^ (middle, LE; right, HE) in response to voltage ramps from –150 to +150 mV before (black trace) and during (red trace) application of capsaicin and after washout (blue trace, left, 1 μM capsaicin; middle, 1 μM capsaicin; right, 100 μM capsaicin) or (**C**) low-pH solution (pH = 4). (**B**) Maximal current density at –60 and +60 mV during application of capsaicin: hTRPV1^WT^ LE, 1 μM capsaicin (*n* = 8), hTRPV1^N331K^ LE, 1 μM capsaicin (*n* = 6), hTRPV1^N331K^ HE, 100 μM capsaicin (*n* = 6) or (**D**) low-pH solution: hTRPV1^WT^ LE, low pH (*n* = 7), hTRPV1^N331K^ LE, low pH (*n* = 6), hTRPV1^N331K^ HE, low pH (*n* = 7). **P < 0.01, by 2-tailed Mann-Whitney *U* test with Bonferroni’s correction for multiple comparisons. Lines indicate the mean.

To investigate the effects of the N331K mutation on TRPV1 function, we generated several clones of tetracycline-regulated (TET-regulated) expression 293 human embryonic kidney (T-REx-293) cell lines stably expressing the mutant TRPV1 (hTRPV1^N331K^) or the WT TRPV1 channel (hTRPV1^WT^ ) (Figure 2A and Supplemental Figure 2A), allowing investigation of the hTRPV1 channel under various expression levels. At high expression (HE) levels (1 μg/mL TET, 21 hours), hTRPV1^WT^-expressing cells became round and detached from the plate surface (Supplemental Figure 2B top right), whereas at low expression (LE) levels (0.1 μg/mL TET, 6 hours), hTRPV1^WT^-expressing cells had normal morphology (Supplemental Figure 2B, top middle). This phenomenon is attributed to cellular Ca^2+^ overload arising from constitutive activity of the overexpressed Ca^2+^-permeable TRPV1 channel (14). Accordingly, we performed functional assays on hTRPV1^WT^-expressing cells under LE conditions. Importantly, hTRPV1^N331K^-expressing cells maintained normal morphology under HE levels (Supplemental Figure 2B, bottom right), suggesting reduced or no constitutive activity of the mutant channels. To evaluate hTRPV1^N331K^ function, we used a fluorescent Ca^2+^ indicator (Fura2-AM) to measure changes in cellular Ca^2+^ during application of the well-known TRPV1 activators capsaicin (2) (Figure 2, B and C), resiniferatoxin (RTX) (15) (Figure 2D and Supplemental Figure 3A), low pH (2) (Figure 2E and Supplemental Figure 3B), tarantula double-knot toxin (DkTx) (16) (Figure 2F and Supplemental Figure 3C), and heat (2) (Figure 2G and Supplemental Figure 3, D and E). Cells expressing hTRPV1^WT^ revealed the typical Ca^2+^ elevation upon application of the various TRPV1 activators (2) (Figure 2, B–G, and Supplemental Figure 3). Strikingly, hTRPV1^N331K^-expressing cells did not show Ca^2+^ elevation in response to the TRPV1 agonists, even under conditions of HE levels and at saturating concentrations of the agonists (100 μM capsaicin, 500 nM RTX, and 5 μM DkTx; Figure 2, B–F, and Supplemental Figure 3, A–C). The heat responses of hTRPV1^N331K^-expressing cells showed a small Ca^2+^ elevation compared with baseline levels, which were comparable to the heat responses observed in naive cells and in hTRPV1^WT^-expressing cells incubated with 20 μM of the TRPV1 inhibitor capsazepine (CPZ) (Supplemental Figure 3, D and E). These results suggest that the above, small heat-induced Ca^2+^ elevation was not mediated by hTRPV1^N331K^ activation. In all cases in which no Ca^2+^ responses were observed, we used the Ca^2+^ ionophore ionomycin as a positive control, which showed the expected rise in cellular Ca^2+^ upon ionomycin application (Figure 2B, Supplemental Figure 2, C and E, Supplemental Figure 3, A–C, and Supplemental Figure 9C). Also, hTRPV1^N331K^-expressing cells of 2 additional clones expressing hTRPV1^N331K^ and cells transfected transiently with hTRPV1^N331K^-mCherry did not show Ca^2+^ elevation in response to application of 100 μM capsaicin (Supplemental Figure 2, C–F), thus verifying that the results were not specific to clonal cell lineage. To further examine the functionality of the mutant hTRPV1 channel, we performed patch-clamp, whole-cell current measurements before, during, and after application of capsaicin (Figure 3, A and B) or low pH (Figure 3, C and D) using voltage ramps. We did not observe a capsaicin or low pH–induced current change in hTRPV1^N331K^-expressing cells, whereas the typical current-voltage relationship was observed for hTRPV1^WT^-expressing cells during application of the agonists (Figure 3, A and C, left).

Together, the results show that potent TRPV1 activators with different mechanisms of action, operating at different sites of the channel (such as protons, capsaicin, and heat) (17, 18), were unable to activate hTRPV1^N331K^, suggesting that the N331K mutation rendered the channel inactive. These results are consistent with the insensitivity to capsaicin of the 2 affected individuals carrying the homozygous N331K mutation (Figure 1B).

### The affected individual reveals no sensitivity to capsaicin, an elevated HPT, and an elevated cold-pain threshold.

**Figure 4:**
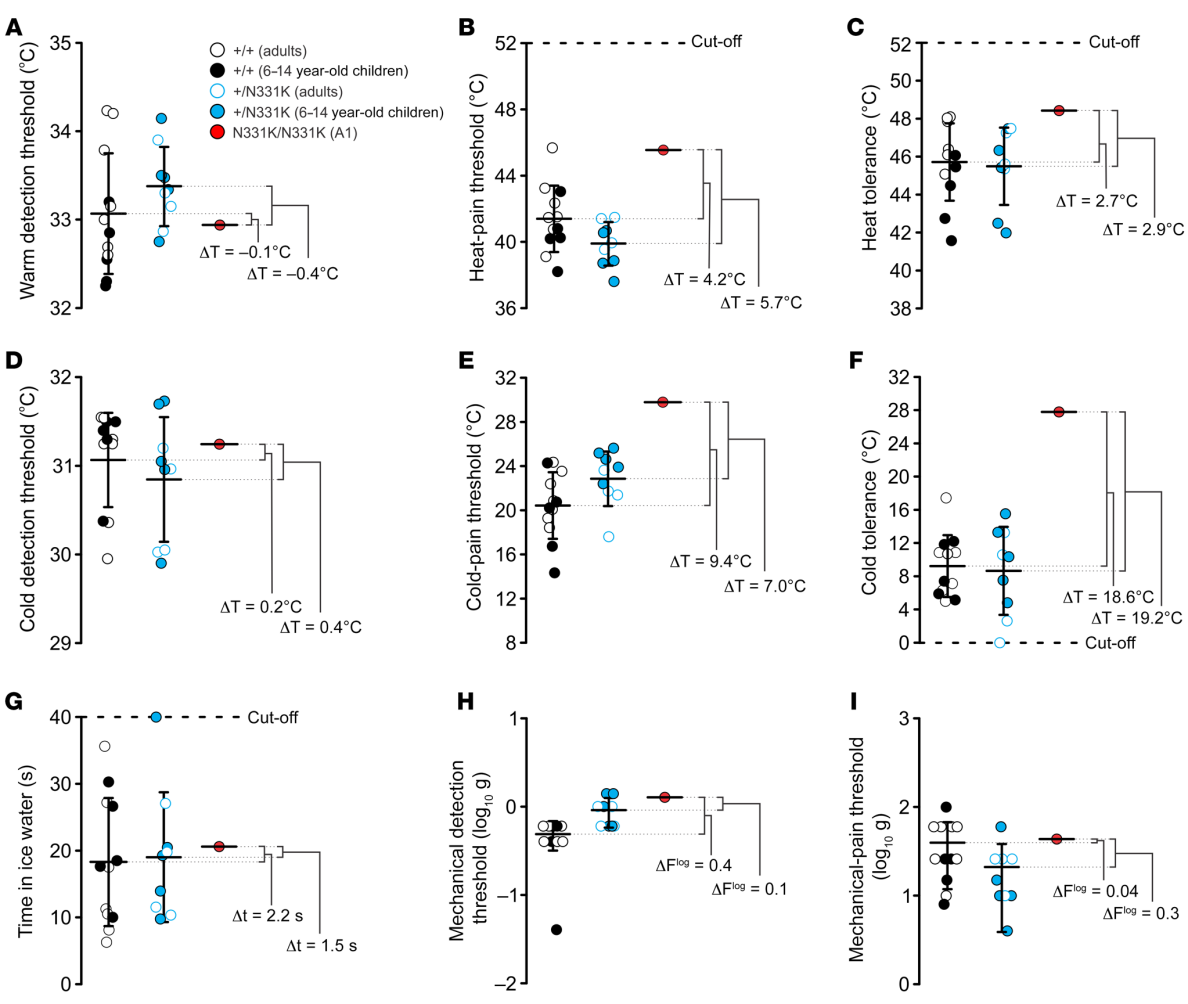
Elevated heat-pain and cold-pain detection thresholds in A1. Thermal and mechanical sensitivity of healthy volunteers (+/+), heterozygous family members (+/N331K), and A1 (N331K/N331K) obtained using QSTs to determine the (**A**) WDT (*n*_+/+_ = 12, *n*_+/N331K_ = 9, mean of *n*_N331K/N331K_ = 13 repeated trials); (**B**) HPT (*n*_+/+_ = 12, *n*_+/N331K_ = 9, mean of *n*_N331K/N331K_ = 31 repeated trials); (**C**) HT (*n*_+/+_ = 12, *n*_+/N331K_ = 9, mean of *n*_N331K/N331K_ = 9 repeated trials); (**D**) CDT (*n*_+/+_ = 12, *n*_+/N331K_ = 9, mean of *n*_N331K/N331K_ = 9 repeated trials); (**E**) CPT (*n*_+/+_ = 12, *n*_+/N331K_ = 9, mean of *n*_N331K/N331K_ = 19 repeated trials); (**F**) CT (*n*_+/+_ = 8, *n*_+/N331K_ = 9, mean of *n*_N331K/N331K_ = 9 repeated trials); (**H**) MDT (*n*_+/+_ = 12, *n*_+/N331K_ = 9, mean of *n*_N331K/N331K_ = 10 repeated trials); and (**I**) MPT (*n*_+/+_ = 12, *n*_+/N331K_ = 9, mean of *n*_N331K/N331K_ = 10 repeated trials). (**G**) Cold pressor test (*n*_+/+_ = 12, *n*_+/N331K_ = 9, mean of *n*_N331K/N331K_ = 4 repeated trials). Data indicate the mean ± SD. Trials are an average of 3–5 repeated measurements. ΔT, Δt, and ΔF^log^ indicate the temperature, time, and force (on a logarithmic scale) differences, respectively, between 2 averaged group results.

A wide range of cells and tissues express TRPV1 (19) and a wide array of functions have been attributed to this channel. Therefore, we set out to examine the affected individuals carrying the N331K mutation in hTRPV1 for clinical anomalies. Since TRPV1 is a heat-activated channel and has been implicated in heat sensing and thermoregulation in animal models and humans (19), we performed quantitative sensory tests (QSTs) with prior knowledge of the genotypes. The tests were performed on the elder A1, as the younger A2 was too young to allow reliable QST measurements. Specifically, we compared the temperature and mechanical sensitivity of A1 with heterozygous family members (E13–E21), healthy volunteers (E1–E12) (some of whom were age-matched 6- to 14-year-old), and a large volume of data from the literature (20, 21). We found similar values for WDT, cold detection threshold (CDT), mechanical detection threshold (MDT), and mechanical-pain threshold (MPT) among the various groups (Figure 4, A, D, H, and I). In contrast, both the heat-pain threshold (HPT) and heat tolerance (HT) values for A1 were elevated relative to the those of control groups (Figure 4, B and C). Surprisingly, his cold-pain threshold (CPT) and cold tolerance (CT) values were largely elevated in comparison with those of the control groups (Figure 4, E and F, and Supplemental Figure 4, C and D). Interestingly, measurements of CT using the cold pressor test were similar for A1 and members of the control groups (Figure 4G). It should be noted that 1 of the healthy volunteers demonstrated an average HPT similar to that of A1 (Figure 4B and Supplemental Figure 4A). Moreover, several healthy volunteers and heterozygous family members reached an average HT value similar to that of A1 (Figure 4C and Supplemental Figure 4B). In addition, the mean HPT for A1 was within the range of healthy humans upon comparison with data from the literature (20, 21). Nevertheless, the scatter of the individually measured points for A1 revealed a group of HPT and HT measurements of high temperature that were not observed in any of the control groups (Supplemental Figure 4, A and B). We conclude that a loss of hTRPV1 function affects both heat pain and cold pain detection thresholds.

Mutations affecting pain sensitivity of the skin may also affect skin structure. We, therefore examined A1’s skin for gross abnormalities by performing a biopsy followed by skin histology and immunohistochemistry. Normal skin structures were observed in A1, including the presence of small afferent nerve fibers in the stratum spinosum, arrector pilus, and sweat glands (Supplemental Figure 5).

**Figure 5:**
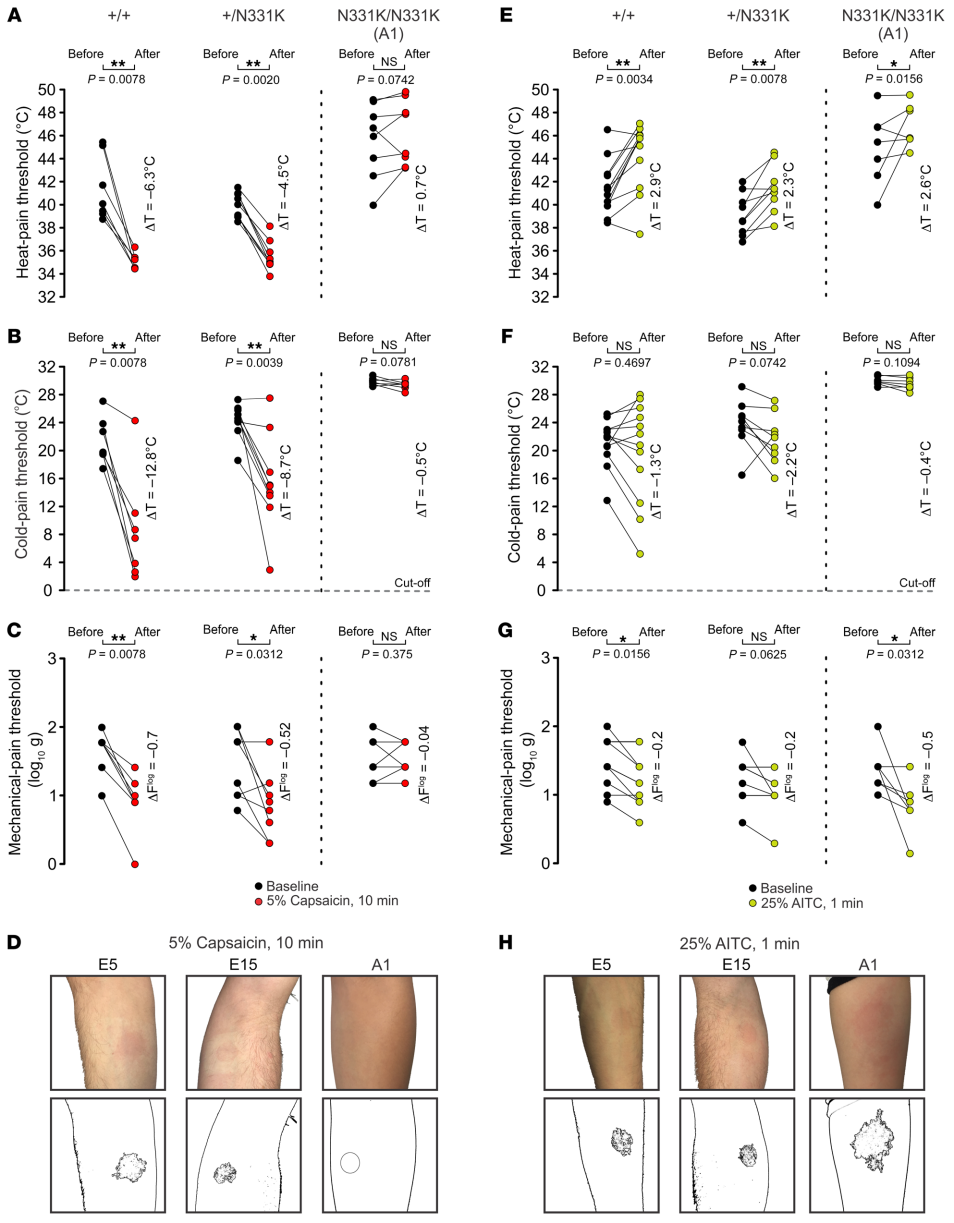
AITC but not capsaicin induces neurogenic inflammation in A1. (**A**–**C**) QST measurements of (**A**) HPT (*n*_+/+_ = 7, *n*_+/N331K_ = 9, *n*_N331K/N331K_ = 8 repeats), (**B**) CPT (*n*_+/+_ = 7, *n*_+/N331K_ = 9, *n*_N331K/N331K_ = 7 repeats), and (**C**) MPT (*n*_+/+_ = 7, *n*_+/N331K_ = 9, *n*_N331K/N331K_ = 7 repeats) before (black dots) and after (red dots) topical application of 5% (w/v) capsaicin to the forearm for 10 minutes (left, healthy volunteers; middle, heterozygous family members; right, A1). Note that the healthy volunteers and heterozygous family members showed thermal heat allodynia, cold hypoalgesia, and mechanical allodynia, whereas A1 did not show any of these phenomena. *P < 0.05 and **P < 0.01, by 1-tailed Wilcoxon signed-rank test. (**E**–**G**) QST measurements of (**E**) HPT (*n*_+/+_= 12, *n*_+/N331K_ = 9, *n*_N331K/N331K_ = 7 repeats), (**F**) CPT (*n*_+/+_ = 12, *n*_+/N331K_ = 9, *n*_N331K/N331K_ = 7 repeats), and (**G**) MPT (*n*_+/+_ = 12, *n*_+/N331K_ = 9, *n*_N331K/N331K_ = 7 repeats) before (black dots) and after (green dots) topical application of 25% (v/v) AITC to the forearm for 1 minute (left, healthy volunteers; middle, heterozygous family members; right, A1). Note that the control groups and A1 showed elevated HPT and minor mechanical allodynia but no change in their CPT. *P < 0.05 and **P < 0.01, by 2-tailed Wilcoxon signed-rank test. (**D** and **H**) Representative images of individual forearms (top) from a healthy volunteer (left), a heterozygous family member (middle), and A1 (right) after topical application of 5% (w/v) capsaicin for 10 minutes (**D**) or 25% (v/v) AITC for 1 minute (**H**) to the forearm using a Finn chamber (for extended data, see Supplemental Figure 6, B and C). Images were processed (bottom) for the extent of the flare using an imaging tool to detect areas of similar tone and color. Note that A1 did not develop a flare after topical application of capsaicin, but developed an extensive flare after topical application of AITC compared with individuals in the control groups. The QSTs were performed 1 minute after removal of the capsaicin or AITC. *P < 0.05 and **P < 0.01, by 1-tailed Wilcoxon signed-rank test (**A**–**C** and **E**–**G**).

Topical application of capsaicin to the skin is known to induce neurogenic inflammation, which is accompanied by the development of local erythema (flare), pain (local burning and stinging sensations), pruritus, thermal and mechanical allodynia, as well as local analgesia (22). In mice, these processes were shown to be mediated by the TRPV1 channel (15, 22). It is therefore expected that capsaicin-mediated neurogenic inflammation would be absent in the affected individuals. To test this prediction, we performed QSTs to determine the HPT, the CPT, and the MPT, before and after topical skin application of 5% (w/v) capsaicin to the forearm for 10 minutes’ duration. The QSTs were performed 1 minute after removing the capsaicin. The healthy volunteers and heterozygous family members showed pronounced flare following capsaicin application (Figure 5D and Supplemental Figure 6B), which was accompanied by ongoing burning pain and local thermal and mechanical allodynia manifested by a reduction in both HPT and MPT (Figure 5, A and C, left and middle). Individuals in the control groups also showed cold hypoalgesia (23), whereby the temperature threshold of cold pain was reduced (Figure 5B, left and middle). In contrast, A1 and A2 reported no painful sensation, nor did they show a flare response following capsaicin application (Figure 5D and Supplemental Figure 6B). Furthermore, A1 showed no significant thermal or mechanical threshold changes in the skin following capsaicin application (Figure 5, A and C).

We conclude that capsaicin-induced neurogenic inflammation was absent in the affected individuals, further supporting the notion that mutation of *TRPV1* causes functional loss of the channel in vivo.

The lack of capsaicin-induced neurogenic inflammation in A1 raises the possibility of a deficiency in his neurogenic inflammatory response. Therefore, we used intradermal histamine administration, which in humans induces local erythema (flare) and swelling (wheal) due to vasodilation and increased vascular permeability, respectively, as well as a sensation of itch and/or pain. The flare response following intradermal histamine injection is mediated mainly by C fibers, which release neuroeffectors promoting vasodilation (24) and can be used to assess loss or dysfunction of small sensory fibers (25). Intradermal injections of 2 histamine doses into the forearm of A1 induced a wheal response and a dose-dependent flare response, which was comparable to the flare responses reported at these doses in the literature (26) and was not observed with intradermal injection of the vehicle (Supplemental Figure 6A). Moreover, injection of histamine led A1 to scratch the area of application and verbally complained that the area became “itchy” and “painful” (Supplemental Table 2), indicating that loss of TRPV1 function did not abolish histamine-mediated itch (27).

### The affected individual reveals extensive neurogenic inflammatory responses to the TRPA1 channel activator allyl isothiocyanate.

Several studies have demonstrated a functional association between TRPA1 and TRPV1 channels (9, 15, 28). To examine whether such an association was altered in A1, we topically applied to his skin allyl isothiocyanate (AITC, mustard oil), a TRPA1 channel activator known to induce a neurogenic inflammatory response (29). In contrast to members of the control group, who reported mild pain, A1 reported extreme pain following a short application of 50% (v/v) AITC and was unwilling to proceed to the standard test duration of 10 minutes. This precluded the collection of sensory data comparable to the data obtained from the literature (30). We therefore reduced the concentration of AITC to 25% (v/v) and applied it for a relatively short period (1 minute). The QSTs were performed 1 minute after removing the AITC. Under these conditions, the pain experienced by A1 was tolerable, and he was willing to perform the sensory tests. In spite of the relatively weak AITC stimulation, our observations indicated that A1 developed a substantial local neurogenic inflammation based on the extent of the flare response and irritation at the point of application. Moreover, following the removal of AITC, A1 scratched the area of application and verbally complained that the area became “itchy” and “painful” (Supplemental Table 2). The healthy volunteers experienced only minor skin irritation and pain following the application of 25% (v/v) AITC, accompanied by a minor flare of the skin (Figure 5H and Supplemental Figure 6C). Both the control groups and A1 showed a small, but significant, elevation in their HPT of approximately 2.5°C following AITC application (Figure 5E) and relatively weak, but significant, mechanical allodynia (Figure 5G). The CPT was unaffected in all tested groups (Figure 5F). Furthermore, all tested individuals reported an elevated and sharp local pain sensation at the point of application when reaching the temperature of the HPT. The results suggest that A1 can develop neurogenic inflammation and inflammatory pain through mechanisms that do not involve a functional TRPV1 channel. The results also indicate that A1 has an increased sensitivity to the TRPA1 activator AITC, causing the sensations of itch and pain.

### Genetic analysis of the affected individuals A1 and A2 supports the association between the N331K mutation in TRPV1 and the observed phenotypes.

The phenotypes of A1 described above raised the question of whether they are directly associated with the N331K mutation in TRPV1 or result, at least in part, from other deleterious mutations in the genome of A1. Given that A1 and A2 are descendants of a consanguineous marriage (Figure 1A), we assumed a recessively inherited, rare causal allele. We therefore performed whole-exome DNA sequencing of A1, A2, examined individuals 13 and 14 (E13 and E14, the parents of A1, Figure 1A). As expected, this analysis disclosed the aforementioned variant c.993C>G (p.N331K) at the *TRPV1* gene, with A1 and A2 being homozygous for the variant, whereas E13 and E14 were heterozygous for the variant. The mutation was not found in the Genome Aggregation Database (gnomAD), which consists of more than 125,000 exome sequences from unrelated individuals. Notably, we did not find in this cohort any homozygous variant in the *TRPV1* gene at this location. The exome analysis of A1 revealed several additional genes predicted to carry deleterious mutations (Supplemental Table 1). One of the genes predicted to carry a deleterious mutation, *PROKR1* c.355G>A (p.E119K), had the prospect of being associated with the phenotypes of A1, as deduced from reviewing the GeneCards database and from a detailed survey of the literature (31–33). This homozygous variant of *PROKR1* was also carried by A2. We found no other matches of homozygous deleterious variants between the exome analysis of A1, A2, E13, and E14. Hence, the exome analysis revealed an additional possible candidate, *PROKR1* c.355G>A, that is probably associated with the phenotypes of A1.

Prokineticin receptor 1 (PROKR1) is a GPCR that is expressed, along with others, in small dorsal root ganglia (DRG) neurons coexpressing the TRPV1 channels. Evidence for the involvement of PROKR1 in nociceptive signaling and inflammatory hyperalgesia comes mainly from studies of the specific naturally occurring peptide agonist Bv8 and from PROKR1-KO mice (31, 32). Mice lacking PROKR1 showed several results that were qualitatively similar to those obtained with TRPV1-KO mice. These included reduced inflammatory heat hyperalgesia following application of mustard oil and reduced sensitivity to noxious heat and acid (31). Interestingly, sensitivity to capsaicin was also largely reduced, but not abolished, in PROKR1-KO mice. Moreover, mice lacking TRPV1 showed a reduced pain response to application of the PROKR1 agonist Bv8, further supporting an interaction between PROKR1 and TRPV1 in nociception and inflammatory pain. Hence, since both A1 and A2 carry identical homozygous mutations of *TRPV1* and *PROKR1*, it raises the question of whether 1 of these 2 mutations, or both, are associated with the phenotypes observed in A1.

As the 2 genes are on different chromosomes, we genotyped most members of the extended family, searching for additional homozygous carriers of the *PROKR1* c.355G>A mutation (Supplemental Figure 7). This search disclosed 2 additional homozygous carriers of the *PROKR1* mutation who were heterozygous carriers of the *TRPV1* mutation. We performed a taste perception test (Figure 1B and Supplemental Figure 1E) and QST measurements (Figures 4 and 5, and Supplemental Figure 4) on 1 of the carriers, E21, as the other did not allow reliable psychophysical measurements. We found that E21 showed temperature and mechanical sensitivity levels similar to those of the control groups (Figure 4 and Supplemental Figure 4). Specifically, the HPT and CPT as well as the HT and CT were similar to those observed in the control groups and different from those of A1 (Figure 4 and Supplemental Figure 4). Furthermore, E21 was sensitive to capsaicin in both the mouth (Figure 1B) and skin (Supplemental Figure 6B) and showed a capsaicin-mediated neurogenic inflammatory response (Figure 5, A–C). The skin flare response of E21 following topical AITC application was mild (Supplemental Figure 6C) and was accompanied by a mild itch sensation, which resembled that experienced by the control groups. In conclusion, E21, a homozygous carrier of the *PROKR1* c.355G>A mutation, did not show the observed phenotypes of A1 and was generally healthy (Supplemental Table 2). Hence, the overall genetic and phenotypic analysis suggests that the phenotypes of A1 are associated with the c.993C>G mutation in *TRPV1* and not with the c.355G>A mutation in *PROKR1*.

We also performed a medical examination and clinical tests together with a structured interview of the parents regarding the health and behavior of A1, A2, and family members, with an emphasis on their sensitivity to painful stimuli and inflammatory pain. A1’s blood sugar and HbA1C levels were within the normal range, suggesting that A1 is not diabetic. The body temperature of both A1 and A2 was maintained at normal values throughout the day. Furthermore, extensive sweating of both A1 and A2, which was not observed in other family members, was reported during the first medical interview. However, during a follow-up medical interview performed a year later, A1 still reported sweating extensively mostly in the face and armpits, while an improvement in the sweating condition was reported for A2 (Supplemental Table 2). The medical history for A1 suggested no gross indication of abnormal bladder function. TRPV1 is known to be expressed in several cell types of the immune system (5). We therefore performed an immunophenotyping test in A1 and 1 heterozygous family member (E17). The comparison showed a similar profile, with a slight increase in γδ T cells (TCRγδ) in A1 (Supplemental Table 2).

In conclusion, the results of the interview and the clinical tests showed that both A1 and A2 were generally healthy, with normal pain and itch perception but some abnormalities related to thermosensitivity for A1.

### The hTRPV1^N331K^ channel subunits assemble properly and reach the plasma membrane.

**Figure 6:**
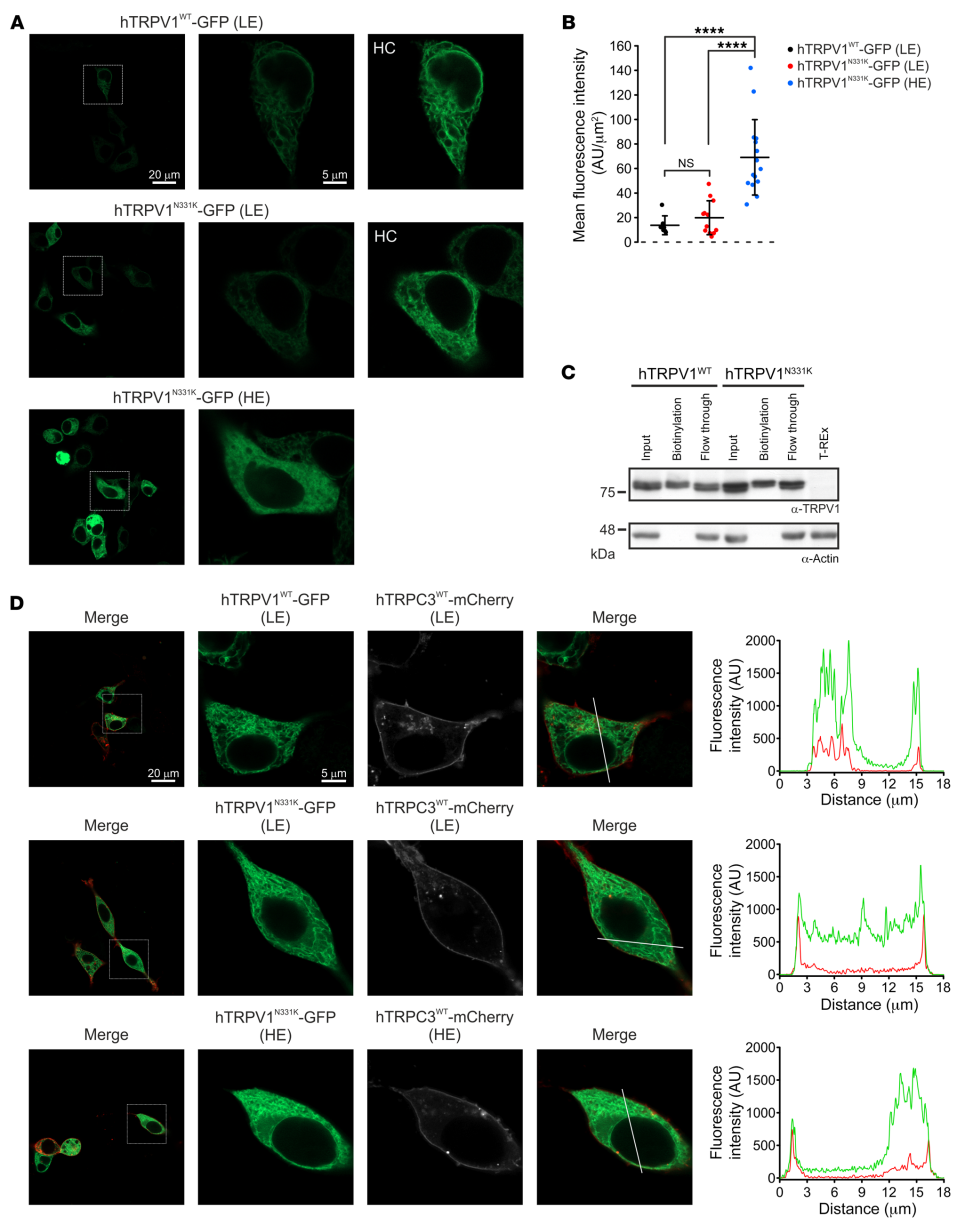
Cellular localization of the hTRPV1^N331K^ channel. (**A**) Representative confocal images of T-REx-293 cells expressing hTRPV1^WT^-GFP at LE levels or hTRPV1^N331K^-GFP at LE or HE levels. Left: Low-magnification (scale bar: 20 μm); middle and right, high-magnification (scale bar: 5 μm, the scale bar is applicable for the middle and right images); and right, high-magnification images at high contrast (HC). Note that the cellular distribution of hTRPV1^WT^-GFP is similar to that of hTRPV1^N331K^-GFP. (**B**) Mean fluorescence intensity measurements from high-magnification images of T-REx-293 cells expressing hTRPV1^WT^-GFP at LE (*n* = 7), hTRPV1^N331K^-GFP at LE (*n* = 12), and hTRPV1^N331K^-GFP at HE (*n* = 15). ****P < 0.0001, by 2-tailed Mann-Whitney *U* test with Bonferroni’s correction for multiple comparisons. Data indicate the mean ± SD. (**C**) Western blot analysis of T-REx-293 cells stably expressing hTRPV1^WT^ or hTRPV1^N331K^ after cell-surface biotinylation, using an anti-TRPV1 antibody (see Methods). Note that both hTRPV1^WT^ and hTRPV1^N331K^ show surface membrane expression (lanes 2 and 5). The intracellular protein actin was used to control the specificity of the assay for cell-surface proteins (*n* = 3). (**D**) Representative confocal images of naive T-REx-293 cells cotransfected with hTRPC3^WT^-mCherry (red or white) together with hTRPV1^WT^-GFP or hTRPV1^N331K^-GFP (green). First column (from left to right): Low-magnification merged images showing hTRPV1-GFP (green) and hTRPC3-mCherry (red). Scale bar: 20 μm (the scale bar is applicable to the first column panels only). Second column: High-magnification view of the boxed area showing hTRPV1-GFP (green). Scale bar: 5 μm (the scale bar is applicable to all panels except for those in the first column). Third column: High-magnification view of the boxed area showing hTRPC3-mCherry (white). Fourth column: High-magnification merged images showing hTRPV1-GFP (green) and hTRPC3-mCherry (red). Fifth column: Profile graphs indicate the fluorescence intensity along the white lines crossing the plasma membrane in the high-magnification merged images.

**Figure 7:**
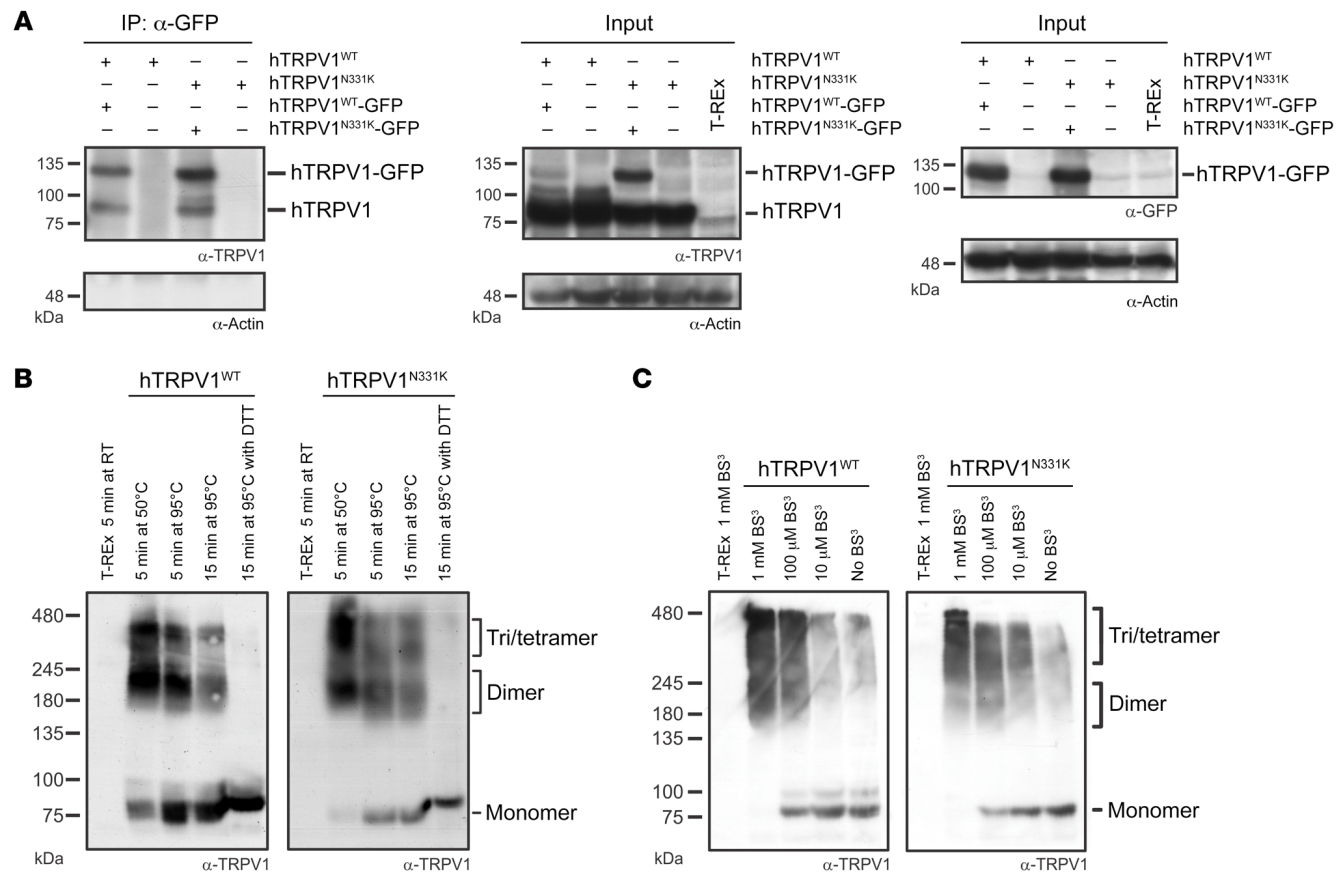
Assembly of the hTRPV1^N331K^ channel. (**A**) T-REx-293 cell lines stably expressing hTRPV1^WT^ and hTRPV1^N331K^ were transfected with hTRPV1^WT^-GFP and hTRPV1^N331K^-GFP, respectively, and immunoprecipitated using an anti-GFP antibody. Western blot analysis using anti-TRPV1 antibody was performed on the immunoprecipitates (left). Western blot analysis using an anti-TRPV1 antibody (middle) or an anti-GFP antibody (right) was performed on the total lysate (input). An anti-actin antibody was used as a positive control (*n* = 3). Note that both hTRPV1^WT^ and hTRPV1^N331K^ were pulled down by the anti-GFP antibody only when expressed together with hTRPV1^WT^-GFP and TRPV1^N331K^-GFP, respectively. (**B**) Western blot analysis of total cell lysates from T-REx-293 cells stably expressing hTRPV1^WT^ or hTRPV1^N331K^ in seminative conditions, using an anti-TRPV1 antibody at different temperatures and incubation durations (*n* = 4, see Methods). Note that the intensity of the higher-molecular-weight bands was reduced or disappeared when lysates were incubated at 95°C or when DTT was added, respectively, while the low-molecular-weight band became stronger. (**C**) BS^3^ crosslinker promoted tetramerization in both hTRPV1^WT^ and hTRPV1^N331K^ channels. An anti-TRPV1 antibody was used in Western blot analysis of total cell lysates from T-REx-293 cells stably expressing hTRPV1^WT^ or hTRPV1^N331K^ and incubated with different doses of the crosslinker BS^3^ in seminative conditions (*n* = 3, see Methods). Note that the higher-molecular-weight band intensities increased as the BS^3^ dose increased.

An intriguing question arising from the functional experiments is how a single point mutation located in the ARD can lead to hTRPV1^N331K^ channel inactivation. The failure to activate the hTRPV1^N331K^ channel may arise from protein instability and degradation, cellular mistrafficking, which prevents the channels from reaching the surface membrane, defects in channel subunit assembly, defective gating, or a combination of all of these mechanisms. Western blot analysis under 2 different amounts of TET and incubation durations showed an increase in full-length protein expression levels in both hTRPV1^N331K^- and hTRPV1^WT^-expressing cells. This suggests that the protein was not prematurely degraded (Figure 2A). Confocal microscopic analysis of cells expressing GFP-tagged hTRPV1^N331K^ (hTRPV1^N331K^-GFP) at LE and HE levels and of hTRPV1^WT^-GFP at LE levels showed a similar cellular distribution of hTRPV1^N331K^-GFP and hTRPV1^WT^-GFP, which was similar to previously reported cellular distribution of rat TRPV1-YFP (rTRPV1-YFP) (34), with a pronounced fluorescence signal observed in intracellular compartments and a small fluorescent fraction located along the plasma membrane (Figure 6A). In general, the fluorescence intensity signal was correlated with the induced expression level (Figure 6, A and B), and intracellular puncta were not observed, thus further supporting the notion that no degradation process occurred. To determine whether hTRPV1^N331K^ channels reached the plasma membrane, we performed cell-surface biotinylation. Accordingly, we detected hTRPV1^N331K^ channels in the plasma membrane in a quantity similar to that of hTRPV1^WT^ channels (Figure 6C). We also examined plasma membrane expression of hTRPV1^WT^ and hTRPV1^N331K^ by an additional independent method using superresolution confocal microscopy (35). Accordingly, we cotransfected naive T-REx-293 cells with hTRPV1^WT^-GFP or hTRPV1^N331K^-GFP (Figure 6D and Supplemental Figure 8, green), together with hTRPC3^WT^-mCherry (Figure 6D and Supplemental Figure 8, white or red), which is predominantly expressed at the plasma membrane (36). Cells coexpressing both hTRPV1 and hTRPC3 channels showed a predominant GFP fluorescence signal across the cell body in intracellular compartments, whereas the mCherry fluorescence signal was observed predominantly at the plasma membrane surrounding the GFP fluorescence signal. However, small stretches within the plasma membrane showed colocalization of both GFP and mCherry fluorescence signals. This was a consistent observation, irrespective of the N331K mutation or the expression level of the hTRPV1^N331K^ channel. Fluorescence intensity line profile analysis across the cells affirmed the presence of an overlap between the GFP and the mCherry fluorescence signals, indicating that TRPV1^WT^ and TRPV1^N331K^ at LE and HE levels reached the plasma membrane (Figure 6D and Supplemental Figure 8). We also performed an immunoprecipitation assay using hTRPV1-expressing cells cotransfected with hTRPV1-GFP to test the ability of hTRPV1^N331K^ channel subunits to assemble into multimers. Accordingly, both hTRPV1^WT^ and hTRPV1^N331K^ proteins were pulled down by GFP antibodies, indicating that the hTRPV1^N331K^ channel subunits preserved the ability to form multimeric channels (Figure 7A). In addition, the use of mildly denaturing gel electrophoresis conditions allowed molecular mass determination of multimeric proteins (37). The results showed that the oligomeric structures of hTRPV1^N331K^ were similar to that of hTRPV1^WT^ (Figure 7B). To further examine hTRPV1^N331K^ subunit assembly into a tetrameric channel, we used a soluble, noncleavable crosslinker, BS^3^, which was previously shown to promote TRPV1 channel oligomerization (37). Incubation of hTRPV1^WT^ with BS^3^ revealed a concentration-dependent formation of dimers, trimers, and tetramers in a manner similar to that of hTRPV1^N331K^ (Figure 7C). In conclusion, these results suggest that hTRPV1^N331K^ was normally assembled and reached the plasma membrane.

### The N331K mutation interferes with the gating process of the TRPV1 channel.

**Figure 8:**
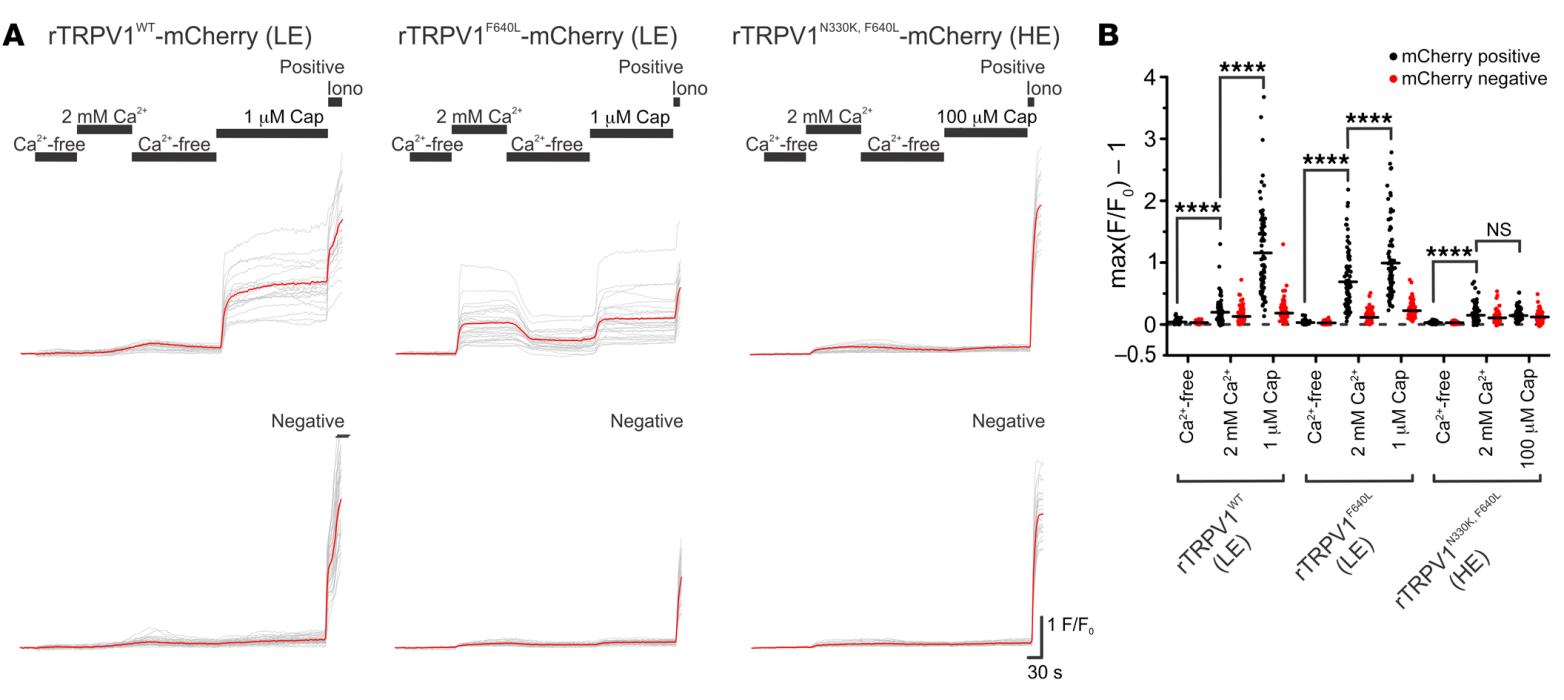
The constitutively active rTRPV1^F640L^ channel is rendered inactive on the N330K background. (**A**) Representative traces of Fura-2–based Ca^2+^ imaging of T-REx-293 cell transiently transfected with rTRPV1^WT^-mCherry (left, LE), rTRPV1^F640L^-mCherry (middle, LE), and rTRPV1^N330K,F640L^-mCherry (right, HE) in response to the Ca^2+^-free extracellular solution, SES (which contains 2 mM Ca^2+^) and application of 1 μM or 100 μM capsaicin in the SES as indicated. (**B**) Maximal normalized fluorescence changed from baseline under the various conditions as indicated: rTRPV1^WT^ LE, mCherry-positive (*N* = 4 plates, *n* = 85 cells); rTRPV1^WT^ LE, mCherry-negative (*N* = 4, *n* = 147); rTRPV1^F640L^ LE, mCherry-positive (*N* = 4, *n* = 83); rTRPV1^F640L^ LE, mCherry-negative (*N* = 4, *n* = 125); rTRPV1^N330K,F640L^ HE, mCherry-positive (*N* = 4, *n* = 123); and rTRPV1^N330K,F640L^ HE, mCherry-negative (*N* = 4, *n* = 133). ****P < 0.0001 and NS *P* > 0.05, by paired, 2-tailed Student’s *t* test with Bonferroni’s correction for multiple comparisons.

To investigate whether the N331K mutation interferes with the gating process of the TRPV1 channel, we examined the effect of the homologous mutation N330K in rTRPV1 (Supplemental Figure 9, A–C) on the background of the F640L mutation in rTRPV1 (rTRPV1^F640L^), which renders the channel constitutively active (14). The F640 residue is located at the C-terminal of the pore helix adjacent to the selectivity filter of the channel and was suggested to be a crucial part of the gating apparatus (14). Therefore, we constructed double-mutant rTRPV1^N330K,F640L^ conjugated to an mCherry fluorescent tag with the aim of identifying transfected cells that express the channel, while enabling the performance of Ca^2+^ imaging. Similar to the results obtained with cells expressing hTRPV1^N331K^ or hTRPV1^N331K^-mCherry (Figure 2, B and C, and Supplemental Figure 2, E and F), rTRPV1^N330K^-mCherry–expressing cells did not show elevation of intracellular Ca^2+^ upon application of 100 μM capsaicin (Supplemental Figure 9, B and C, right). Cells expressing rTRPV1^WT^-mCherry showed only a small elevation of intracellular Ca^2+^ upon replacement of Ca^2+^-free with Ca^2+^-containing solution in the absence of an agonist and a large elevation of intracellular Ca^2+^ upon application of 1 μM capsaicin. Cells expressing rTRPV1^F640L^-mCherry showed a large elevation of intracellular Ca^2+^ upon replacement of Ca^2+^-free with Ca^2+^-containing solution in the absence of an agonist, demonstrating that these channels were constitutively active. We observed a slightly larger elevation of intracellular Ca^2+^ upon application of 1 μM capsaicin (14). Strikingly, cells expressing rTRPV1^N330K,F640L^-mCherry showed only a slight elevation of intracellular Ca^2+^ upon replacement of Ca^2+^-free with Ca^2+^-containing solution in the absence of an agonist and during application of 100 μM capsaicin (Figure 8, A and B). Hence, the N330K mutation suppressed the large constitutive activity rendered by the F640L mutation on the rTRPV1 channel, suggesting the involvement of the N330 residue in the activation process of the TRPV1 channel.

The results of this work extend previous functional roles attributed to the ARD of TRPV1 (12, 38) by showing that a missense mutation in a conserved residue of the ARD disrupted the gating processes of the channel.

## Discussion

This study constitutes what we believe to be the first comprehensive characterization of humans carrying a homozygous missense mutation in the *hTRPV1* gene (Supplemental Figure 1A) that renders the channel nonfunctional. The conclusion that the TRPV1 channel is nonfunctional was derived from experiments demonstrating the inability of different TRPV1 activators, acting at different sites of the protein, to open the channel in vitro (Figures 2 and 3) and the insensitivity of the affected individuals to application of capsaicin to the mouth (Figure 1B) and skin (Figure 5, A–D) in vivo. Experiments designed to examine the expression level, subunit assembly, and cellular localization of the mutant TRPV1 channel showed results similar to those obtained with WT TRPV1, suggesting that the functional loss of the channel is not a consequence of impairment in the expression, cellular trafficking, or protein assembly (Figures 6 and 7) in vitro. Quantitative mRNA measurement of *TRPV1* extracted from PBMCs of A1 and control group individuals showed similar expression levels, suggesting that the mutation does not affect mRNA *TRPV1* expression levels in vivo (Figure 1C). The severity and extent to which the N331K mutation affected the activation process of the channel was further demonstrated by a constitutive activity-inducing mutation in TRPV1, located at the pore helix of the channel, which was unable to rescue the function of the mutant channel (Figure 8). Together, these results suggest that the N331K mutation rendered the channel nonfunctional by disrupting a necessary step in the activation process of the channel, revealing a pivotal functional role of the ARD region comprising the N331 locus in the activation process of TRPV1. The ARD was previously suggested to play roles in channel assembly, protein-protein interaction, and modulation of channel activity (39, 40), but only recently in channel activation. Indeed, several recent structural simulation studies have suggested an essential role of the ARD of TRPV1 (41, 42) and TRPV3 (43) channels during the activation process. Hence, it may be worthwhile to further investigate the mechanism by which the ARD participates in TRPV1 channel activation and examine whether conservation of the N331 locus across the TRPV and TRPC channel subfamilies extends to a functionally conserved role in the activation process of these channels.

Clinically, the lack of functional TRPV1 was not accompanied by a deficit in the response to skin irritants, such as AITC or histamine (Figure 5, E–H, and Supplemental Figure 6), or by a pronounced deficit in any sensory modality other than changes in temperature-dependent thresholds of noxious stimuli (Figure 4 and Supplemental Figure 4), or by the appearance of any clear pathology under normal conditions (Supplemental Table 2). Two main on-target adverse effects were observed during human trials of several TRPV1 antagonists: hyperthermia and thermal hypoethetics. Interestingly, drug tolerance appeared to develop only to the hyperthermic effect. These phenomena are consistent with the normal core body temperature and heat hyposensitivity observed in the affected individual. Importantly, the overall phenotype of the affected individuals is also consistent with studies of TRPV1-KO mice, which reported a loss of capsaicin responsiveness and mild effects on acute noxious heat sensations (15, 44). Surprisingly, QST measurements of A1 revealed elevated CPT and reduced cold pain tolerance using a Peltier thermode, whereas normal cold pain tolerance was measured when using the cold pressor test (Figure 4, E–G, Supplemental Figure 4, C and D, and see Methods). The later result could be explained by the widespread application of cold stimulus in the cold pressor test in comparison with the local cold application using the Peltier thermode. To the best of our knowledge, a loss of TRPV1 function has not been associated previously with changes in the CPT in humans (11), while in mice lacking TRPV1, sporadic conflicting evidence showed either cold hypersensitivity (45) or no change in the CPT (46, 47). In addition to the unusually high cold hypersensitivity, A1 also demonstrated high sensitivity to topical forearm application of AITC, a TRPA1 agonist, revealing extreme aversion at a high concentration and a large flare response at a relatively low concentration (Figure 5H). This finding is consistent with the observation of hypersensitivity to garlic extract in the mouth of an individual carrying a mutation at the second intron of the *TRPV1* gene (9), which induces pungency through activation of the TRPA1 channel (48). However, this result is incompatible with the lack of thermal hyperalgesia observed following AITC application to the hind paws of TRPV1-KO mice (15), raising the possibility of a species-specific mechanism. TRPV1 is expressed in several types of nociceptive neurons and interacts with numerous proteins such as TRPA1 that are expressed in nociceptors. Therefore, the enhanced cold sensitivity of A1 and his hypersensitivity to AITC could be explained by an alteration of the molecular interaction network of TRPV1 with other proteins, particularly the molecular interaction of TRPV1 and TRPA1 (28). Alternatively, the above phenotypes could be explained at the neuronal network level, i.e., differences in the sensory system organization of humans and mice (49, 50), or by a change in the balance between TRPV1-lineage and non-TRPV1-lineage neuronal inputs to the dorsal horn of the spinal cord. Indeed, the latter explanation was supported by a recent study, which examined the activity of dorsal horn spinal cord neurons at different temperatures, demonstrating an interaction between the heat and cold pathways (51) following inflammation or oxaliplatin administration. Accordingly, the authors showed that hypersensitivity to heat is accompanied by cold hyposensitivity, under inflammatory conditions (formalin and PGE2), and that hypersensitivity to cold is accompanied by heat hyposensitivity, following oxaliplatin administration. The authors proposed a push-pull mechanism in processing cold and heat inputs, revealing a synergic mechanism to shift thermosensation following injury. Hence, this synergic mechanism could be at play in the current case, in which the hyposensitivity to heat caused by the lack of TRPV1 function influenced spinal cord input integration, resulting in cold hypersensitivity. Nonetheless, this study supports new roles of the TRPV1 channel, whereby the channel determines a set-point for noxious temperature detection and inflammatory pain.

Parental consanguinity has been associated with an increased risk of autosomal recessive disorders and congenital anomalies in the offspring (52). Since the affected individuals (A1 and A2) are the descendants of a consanguineous marriage (Figure 1A), it was important to perform whole-exome analysis of A1, A2, E13, and E14 (the parents of A1) to examine whether additional genes carrying deleterious mutations (Supplemental Table 1) could be associated with the phenotypes observed for A1. The genetic analysis revealed an additional homozygous gene variant, *PROKR1* c.355G>A (p.E119K), which has a high prospect of being associated with the phenotypes of A1, as deduced from a review of the GeneCards database and from a detailed survey of the literature (31–33). This homozygous variant of *PROKR1* was also carried by A2 (Supplemental Table 1), raising the question of which of the 2 mutated genes, or whether both of them, are associated with the phenotypes of A1. Importantly, an additional family member, who is a homozygous carrier of the *PROKR1* c.355G>A variant and a heterozygous carrier of the *TRPV1* c.993C>G variant (E21, Supplemental Figure 7), was thoroughly examined and did not reveal any of the phenotypes observed in A1 (Figure 1B, Supplemental Figure 1E, Figures 4 and 5, Supplemental Figure 4, and Supplemental Table 2). These findings suggest that the phenotypes of A1 are associated with the *TRPV1* c.993C>G mutation and not the *PROKR1* c.355G>A mutation. However, since no homozygous carrier of *TRPV1* c.993C>G who is not a homozygous carrier of *PROKR1* c.355G>A was identified and examined, it remains possible that the *PROKR1* c.355G>A variant exacerbates the phenotypes observed in A1.

In conclusion, this study provides what to our knowledge is the first direct evidence for pain-related functional changes linked to TRPV1 in humans, which should be considered in the development of TRPV1 antagonists as novel therapeutic pain-relieving agents.

## Methods

Additional details can be found in Supplemental Methods.

### Site-specific mutagenesis

Human *TRPV1* (NM_080706.3) was subcloned into the pJET1.2 cloning vector (see Table 1). All site-directed mutagenesis procedures were performed as follows: PCRs were performed using primers harboring the mutation and a 5′ phosphate modification. PCR products were extracted and cleaned from agarose gel (NucleoSpin, MAN-740609, Macherey Nagel), ligated using the T4 ligase kit (K1422, Thermo Fisher Scientific), and transformed into competent DH5α *E*. *coli*. After confirming the mutation by sequencing, a second PCR was performed using KpnI-f and EcoRI-r or EcoRI-no-stop-r primers (Integrated DNA Technologies [IDT], see Table 2). Consequently, the PCR products were subcloned into a destination plasmid (pCDNA4, pCDNA4-mCherry or pCDNA4-GFP, see Table 1) using KpnI-HF and EcoRI-HF restriction enzymes (R3142, R3101, New England BioLabs). All ORFs were fully sequenced.

Table 1 lists the plasmids used in this study, and Table 2 lists the DNA primers used in this study.

### Generation of stable hTRPV1 T-REx-293 cell lines

To generate stable pCDNA4-hTRPV1^WT^-T-REx-293 and pCDNA4-hTRPV1^N331K^-T-REx-293 cell lines, the pCDNA4-hTRPV1^WT^ and pCDNA4-hTRPV1^N331K^ plasmids were first digested using the NruI restriction enzyme (R0192, New England BioLabs) before transfection. Transfected cells were grown first in medium supplemented with 5 μg/mL blasticidin (ant-bl-1, InvivoGen) for 24 hours and then moved to a medium which also contained 400 μg/mL zeocin (ant-zn-1, InvivoGen). Cell medium was changed every 48–72 hours for several weeks until the appearance of colonies. Colonies were isolated and verified for the expression of hTRPV1^WT^ or hTRPV1^N331K^ using sequencing and Western blot analysis. TRPV1 expression was induced using TET (T7660, MilliporeSigma).

### Cell culturing

T-REx-293 cells (Thermo Fisher Scientific) were grown at 37°C, 5% CO_2_ in DMEM (01-055-1A, Biological Industries) supplemented with 10% TET system–approved FBS (04-005-1A, Biological Industries), 1% penicillin-streptomycin (03-031-1B, Biological Industries), 2 mM l-glutamine (03-020-1B, Biological Industries), and 5 μg/mL blasticidin (ant-bl-1, InvivoGen). For stable pCDNA4-hTRPV1^WT^-T-REx-293 and pCDNA4-hTRPV1^N331K^-T-REx-293 cell lines, the above medium was supplemented with the addition of 100 μg/mL zeocin (ant-zn-1, InvivoGen). Cells were not used above passage 25. Transfections were performed using TransIT-LT1 transfection reagents (MC-MIR-2300, Mirus) according to the manufacturer’s protocol.

#### Stable hTRPV1^WT^ and hTRPV1^N331K^ T-REx-293 cell lines.

To validate the presence of *hTRPV1* mRNA in stable cell lines, hTRPV1 expression was induced using TET (1 μg/mL, 21 hours). Thereafter, cells were washed twice with 5 mL ice-cold PBS and detached using 5 mL ice-cold PBS without Ca^2+^ or Mg^2+^ (02-023-1A, Biological Industries). The cells were collected, followed by centrifugation at 300*g* for 5 minutes, and then the supernatant was removed. The pellet was resuspended in 1 mL TriReagent (Bio-Lab), and RNA was extracted according to the manufacturer’s instructions. Full sequencing of *hTRPV1* cDNA was performed to validate the presence of WT or c.993C>G transcripts.

### Antibodies

The following primary antibodies were used in this study: rabbit polyclonal anti-GFP (MBL, lot: 076); mouse monoclonal anti–β actin antibody (ab8224, lot: GR221876-7, Abcam); rabbit polyclonal TRPV1 antibody against the N-terminal (124-153) (AP13988a, clone ID: RB33721, lot: SA110630AA, clone ID: RB59267, lot: SA170811DD, Abgent); and rabbit polyclonal PGP9.5 antibody (Z5116, Dako). The following secondary antibodies were used in this study: peroxidase AffiniPure goat anti–mouse IgG (H+L) antibody (115-035-003, lot: 139283) and goat anti–rabbit IgG (H+L) antibody (111-035-003, lots: 115298 and 139196). For blotting TRPV1 in immunoprecipitation experiments, the peroxidase IgG fraction monoclonal mouse anti–rabbit IgG, light-chain specific (221-032-171, lot 120918) was used. All secondary antibodies were purchased from Jackson ImmunoResearch.

### Immunoblot analysis

pCDNA4-TRPV1^WT^-T-REx-293, pCDNA4-TRPV1^N331K^-T-REx-293, and T-REx-293 cells were washed twice with 5 mL ice-cold PBS and detached using 5 mL ice-cold PBS (without Ca^2+^ or Mg^2+^) and a scraper. Cells were centrifuged at 300*g* for 5 minutes at 4°C, and the supernatant was removed. The cell pellet was solubilized with 200 μL lysis buffer (1% Triton X-100, 25 mM Tris–HCl [pH = 7.5], 150 mM NaCl, 5 mM EDTA, and protease inhibitor cocktail [1:100, P8340 MilliporeSigma]) and was rotated for 1 hour at 4°C. Lysates were centrifuged at 12,000*g* for 20 minutes at 4°C, and the supernatant was collected. Sample buffer DTT (×5) (330 mM Tris, 500 mM DTT, 10% SDS, 50% glycerol, 0.05% bromophenol) was added, and samples were heated for 5 minutes at 95°C. Samples were separated on an 8% polyacrylamide gel and immunoblotted according to standard procedures (1658037, Mini-PROTEAN, Bio-Rad). For heat-dependent tetramerization experiments in seminative conditions, sample buffer (×2) (100 mM Tris, 4% SDS, 20% glycerol, 0.005% bromophenol) was used. Samples were heated at different temperatures and durations. For DTT treatment, sample buffer DTT (×2) (132 mM Tris, 200 mM DTT, 4% SDS, 20% glycerol, 0.02% bromophenol) was used, and samples were heated for 15 minutes at 95°C. Samples were separated on a 6% polyacrylamide gel and immunoblotted according to standard procedures. For crosslinking tetramerization experiments in seminative conditions, samples were incubated with 10 μM, 100 μM, and 1 mM BS^3^ (S5799, MilliporeSigma) for 30 minutes at room temperature (RT). To end the crosslinking reaction, an equal volume of 1 M glycine was added for 20 minutes at RT. Sample buffer DTT (×5) was added, and samples were heated for 15 minutes at 95°C. Samples were separated on a 6% polyacrylamide gel and immunoblotted according to standard procedures.

### Immunoprecipitation

Stable pCDNA4-hTRPV1^WT^-T-REx-293 and pCDNA4-hTRPV1^N331K^-T-REx-293 cells were transiently transfected with pCDNA4-hTRPV1^WT^-GFP and pCDNA4-hTRPV1^N331K^-GFP, respectively. Lysates were prepared according to the standard protocol. Lysates were centrifuged at 12,000*g* for 20 minutes at 4°C, and the supernatant was incubated with GFP antibody in the presence of protein A/G PLUS agarose (20423, Pierce, Thermo Fisher Scientific) and rotated overnight at 4°C. Immunoprecipitates were washed 5 times with 500 μL lysis buffer, separated on a 8% polyacrylamide gel, and immunoblotted according to standard procedures.

### Surface biotinylation assay

pCDNA4-hTRPV1^WT^-T-REx-293, pCDNA4-hTRPV1^N331K^-T-REx-293 and T-REx-293 cells were collected as described above, and a surface biotinylation assay was performed according to the protocol provided in the Pierce cell-surface protein isolation kit (89881l, Thermo Fisher Scientific).

### Ratiometric calcium imaging

Cells were seeded on 35 mm plates (430165, Corning) coated with polylysine (P8920, MilliporeSigma) or on a chambered coverslip (80826, Ibidi) and loaded with fura-2 AM dye (F-1221, Molecular Probes, Thermo Fisher Scientific) at a final concentration of 2 μM in clean DMEM for 45 minutes, followed by washing and incubation in clean DMEM or Ca^2+^-free solution (Table 4 E6) for further 45 minutes. Plates were mounted on an inverted microscope (Nikon, Eclipse Ti) equipped with an Exi Aqua monochromator (QImaging). Perfusion was performed at 2 mL/min. The ratiometric [Ca^2+^]_i_ was measured by exciting the cells at 340 and 380 nm (Lambda DG4, Sutter Instruments) and measuring the emission at 510 nm (Filter set 79001, Chroma). Images were taken every 1 second, monitored online, and then analyzed offline using Nikon Elements AR Software (Nikon). All values were normalized to the value at *t* = 0 (F_340/380_/F^0^_340/380_). The maximal normalized fluorescence (F) change from baseline during application was used for analysis [max(F_340/380_/F^0^_340/380_) – 1].

### Electrophysiology

Cells were seeded on polylysine-coated 16 mm coverslips. Whole-cell currents were recorded at RT using borosilicate patch pipettes of 3–5 MΩ resistance. Electrical signals were amplified using Axopatch 1D (Axon Instruments), and data were captured using a Digidata 1440A (Molecular Devices) interfaced to a computer. The membrane potential was held at 0 mV, and currents were measured in response to 1,050 ms voltage ramps from –150 to +150 mV every 5 seconds.

### Confocal imaging

Images of cells were acquired using a confocal microscope (Zeiss LSM 980 with Airyscan 2) with a Zeiss Plan Apochromat ×63/1.4 oil DIC M27 objective controlled by ZEN software (version 3.3 blue). Cells were seeded on 35 mm plates (FD-35-100, World Precision Instruments [WPI]) coated with polylysine. Confocal images were taken of T-REx-293 cells transfected with pCDNA4-hTRPV1^WT^-GFP or pCDNA4-hTRPV1^N331K^-GFP. Laser irradiation at 488 nm was used to excite the GFP and imaged using a 490–550 nm band-pass emission filter. Detector gain and laser intensity were constant for all experimental groups. Superresolution images were taken from T-REx-293 cells cotransfected with either pCDNA4-hTRPV1^WT^-GFP or pCDNA4-hTRPV1^N331K^-GFP together with pCDNA4-hTRPC3^WT^-mCherry using the Airyscan mode to achieve a resolution of up to 140 nm. Laser irradiation at 488 nm and 561 nm was used to excite the GFP and mCherry, respectively. GFP was imaged using 420–480 nm and 495–550 nm band-pass emission filters, and mCherry was imaged using a 574–720 nm band-pass emission filter.

### Solutions

The following chemicals were used in the intracellular and extracellular solutions (for details, see Table 3 and 4, respectively): CsMeSO_3_ (C1426), CsCl (289329), CsOH (C8518), NaCl (7548-4400, DAEJUNG), MgCl_2_ (M9272), MgSO_4_ (6070-12, Mallinckrodt Chemicals), sodium l-glutamate (49621), KCl (P5405), CaCl_2_ (C7902), EGTA (E4378), MgATP (A9187), NaGTP (G8877), HEPES (H3375), citric acid (C2404), NaOH (7708-10, Mallinckrodt Chemicals), H_2_SO_4_ (339741), HCl (H1758), and d-glucose (1916-01, J.T. Baker). All chemicals were purchased from MilliporeSigma unless otherwise indicated.

### Pharmacology

The following pharmacologic agents were used: capsaicin (M2028, MilliporeSigma), RTX (RTX, 1137, Tocris), DkTx (provided by A. Priel, The Institute for Drug Research [IDR], School of Pharmacy, Faculty of Medicine, The Hebrew University of Jerusalem, Jerusalem, Israel), and AITC (377430, MilliporeSigma).

### Quantitative sensory testing

Thermal quantitative sensory testing was performed using a Peltier thermode (30 × 30 mm), placed on the volar side of the forearm, and a TSA 2001-II device (Medoc). Participants were first tested for WDT and CDT using the method of level. Baseline temperature was set at 32°C. The temperature changed, and participants were asked to respond “YES” or “NO,” depending on whether or not they perceived a thermal sensation. A YES response led to a smaller stimulus, whereas a NO response led to a larger stimulus. Thermal thresholds were calculated as the averages of several trials.

The HPT and CPT were tested using the method of limits before and after application of 25% AITC (v/v) diluted in olive oil or 5% capsaicin (w/v) diluted in 50% ethanol (young children were not tested with topical application of capsaicin). Both the AITC and capsaicin were applied on the volar side of the forearm using a filter paper (AL5311GEN, Gamida) soaked with 200 μL of the solution placed in a Finn chamber (AL4321GEN, Gamida). The QSTs were performed 1 minute after removing the filter paper soaked with capsaicin or AITC. The baseline temperature was set at 32°C, and the ramp temperature changed at 1°C/s. The cutoff temperatures were 52°C and 0°C for HPT and CPT, respectively. The intensity of the stimuli was increased until the participant perceived thermal pain, at which point they pressed a button, which stopped the ramp and returned the heat to baseline. The instructions for CPT/HPT were “Press the button when you start to feel heat/cold pain.” HT and CT tests were performed similarly to the HPT and CPT tests with the instruction, “Press the button when the pain is not tolerable.” Measurement of CT using the cold pressor test was performed by dipping the forearm in ice water and measuring the time until it was not tolerable. The cutoff time was 40 seconds. The MDT and MPT were measured at the anterior forearm using von Frey monofilaments (VFMs), with the constant length and increasing force ranging from 0.008–300*g* (Stoelting). The VFMs were forced perpendicularly to the skin surface until a bending of 3–5 mm was produced. The VFMs were held in this position for approximately 2 seconds, with a 20-second break given between 2 successive stimuli. The measurements were performed before and after topical application of capsaicin (5%, w/v) or AITC (25%, v/v) in a randomized sequence until the MDT and MPT were determined. Examinees kept their eyes closed during the investigation to avoid visual feedback concerning the stimuli. The patients were asked to give a clear verbal signal if the stimulus was or was not perceived (MDT) and if the perceived signal was painful (MPT).

All psychophysical tests were performed with prior knowledge of the genotype.

### Statistics

Statistical analyses were conducted using GraphPad Prism 8 (GraphPad Software) or SPSS Statistics (IBM). Statistical significance was determined using a paired and unpaired, 2-tailed Student’s *t* tests, 2-way ANOVA, 1- and 2-tailed Mann-Whitney *U* tests, and 1- and 2-tailed Wilcoxon signed-rank tests following Bonferroni’s correction for multiple comparisons. No statistical methods were used to predetermine the sample sizes. A *P* value of less than 0.05 was considered statistically significant. Data represent the mean ± SD.

### Study approval

The affected individuals (A1 and A2) and family members are of Palestinian Arab ethnicity. The proband (A1), an 11-year-old male, is the child of a consanguineous marriage (father, E13; mother, E14) and has 4 brothers and 2 sisters. The second affected individual (A2, female) is the child of a consanguineous marriage (father, E15; mother, E16) and has 1 brother and 2 sisters. The fathers of A1 and A2 are brothers, and the 2 mothers are sisters (Figure 1A and Supplemental Table 2). The family was clinically examined at the Hadassah Hospital in Jerusalem, Israel. The heterozygous volunteers (E13–E21) are family members, and the healthy volunteers (E1–E12) were recruited from the Hebrew University Medical School of Jerusalem, Israel. Written informed consent was obtained from all participants over the age of 18 years and from the family guardian of participants under the age of 18 years prior to inclusion in the study. All participants under 18 years of age were escorted by their parents. Male and female participants were eligible if they did not have any skin disease or allergies to capsicum, mustard plants, or other products. Healthy volunteers had to be in good health and taking no medications. The study was approved by the ethics committee of Hadassah Hospital (0641-16-HMO) and conducted according to the principles of the Declaration of Helsinki and the Edinburgh 2000 revisions (Appendix 5) and the ICH Harmonized Tripartite Guidelines for Good Clinical Practice, FDA Code of Federal Regulations (Sections 21:56, 57, and 45 CFR 164).

## Author contributions

BK and RZ established the strategy of the experiments; performed the QSTs and the electrophysiological, biochemical, cloning, mutagenesis, and imaging experiments; analyzed and interpreted the data; designed the structured interview with the parents; prepared the figures; and drafted and wrote the manuscript. SE maintained communication with the Palestinian family during the entire project, performed the clinical examinations, and supervised the QSTs. CM identified the Palestinian family in her clinic at Hadassah Hospital, arranged the initial genetic test, and performed preliminary clinical examinations. OE sequenced the mutant *hTRPV1* gene and performed and analyzed the whole-exome sequencing. SL performed imaging experiments. ED provided the use of the Pain Clinic in Hadassah Hospital and established and supervised the QSTs. MP performed cloning and mutagenesis. SKE performed the immunological tests. EB performed the skin histology. SG communicated with A1 and family members during the QST, maintained the communication with family members, and helped in the structured interview of the parents. AMB established and maintained communication with the Pain Clinic in Hadassah Hospital, participated in the design of the experiments, interpreted the data, and drafted and reviewed the manuscript. BM initiated the project, supervised the experiments, interpreted the data, and drafted and wrote the manuscript. The method used in assigning the co–first authorship order was based on the relative contributions of the authors to the manuscript.

## Supplementary Material

Supplemental data

ICMJE disclosure forms

## Figures and Tables

**Table 1:**
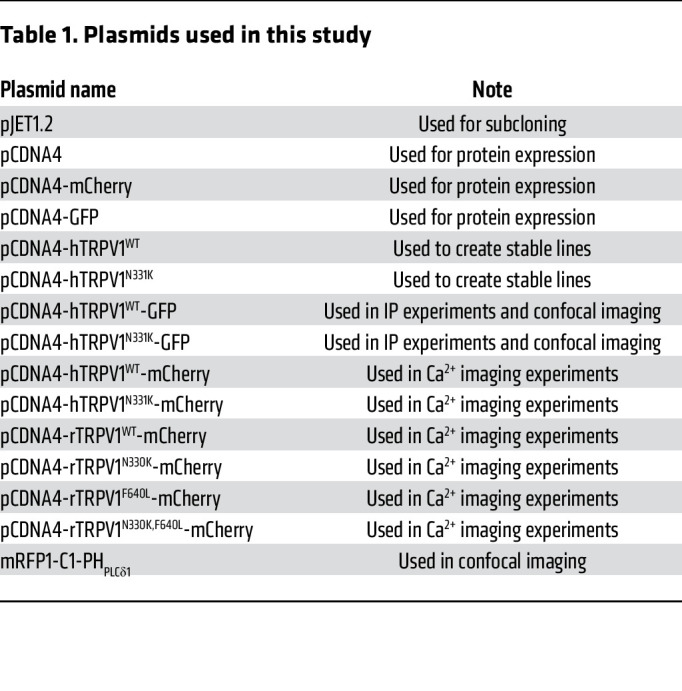
Plasmids used in this study

**Table 4:**
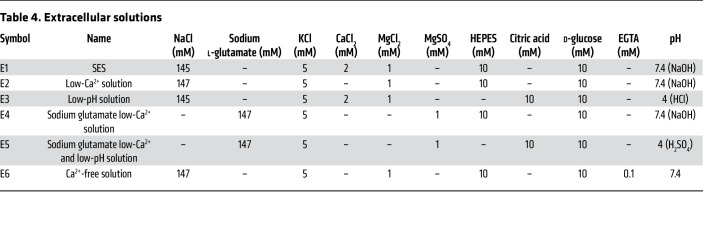
Extracellular solutions

**Table 3:**
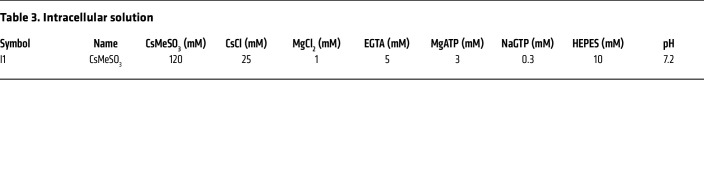
Intracellular solution

**Table 2:**
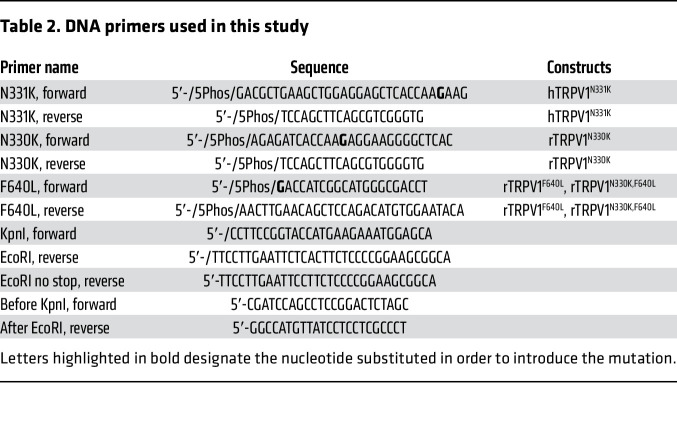
DNA primers used in this study

## Version 1 12/01/2022

In-Press Preview

## Version 2 02/01/2023

Electronic publication
