# Addendum to Nociception and pain in humans lacking a functional TRPV1 channel

**DOI:** 10.1172/JCI205878

**Published:** 2026-04-01

**Authors:** Ben Katz, Rachel Zaguri, Simon Edvardson, Channa Maayan, Orly Elpeleg, Shaya Lev, Elyad Davidson, Maximilian Peters, Shlomit Kfir-Erenfeld, Esther Berger, Shifa Ghazalin, Alexander M. Binshtok, Baruch Minke

Original citation: *J Clin Invest*. 2023;133(3):e153558. https://doi.org/10.1172/JCI153558

Citation for this addendum: *J Clin Invest*. 2026;136(7):e205878. https://doi.org/10.1172/JCI205878

Following the publication of their article, the authors noted that the description of the cold phenotype in an individual lacking functional TRPV1 due to a homozygous missense mutation in the *TRPV1* gene has been misinterpreted in some articles citing this work. For clarity, sentences in the abstract, Results, and Discussion have been updated to better describe the cold phenotype as shown below. The HTML and PDF versions of the paper have been updated.

Abstract

Furthermore, quantitative sensory testing of A1 revealed an elevated heat-pain threshold but also, surprisingly, marked cold hypersensitivity, with cold pain reported at temperatures that are innocuous to healthy individuals, and extensive neurogenic inflammatory, flare, and pain responses following application of the TRPA1 channel activator mustard oil.

Results

The affected individual reveals no sensitivity to capsaicin, an elevated HPT, and marked cold hypersensitivity, with cold pain reported at temperatures that are innocuous to healthy individuals.

Surprisingly, his cold-pain threshold (CPT) and cold tolerance (CT) values were shifted toward warmer temperatures in comparison with those of the control groups (Figure 4, E and F, and Supplemental Figure 4, C and D).

Discussion

Surprisingly, QST measurements of A1 revealed a shift toward warmer temperatures of CPT and CT using a Peltier thermode, whereas normal cold pain tolerance was measured when using the cold pressor test (Figure 4, E–G, Supplemental Figure 4, C and D, and see Methods).

## Version 1 04/01/2026

Electronic publication

