# Supplementary material for: Nociception and pain in humans lacking a functional TRPV1 channel
Source: J Clin Invest. 2023 Feb 1;133(3):e153558. doi: 10.1172/JCI153558 (PMC9888381; doi:10.1172/JCI153558)
Supplement: Supplemental data [file jci-133-153558-s225.pdf]

Supplemental Information for

Nociception and pain in humans lacking functional TRPV1 channel

Ben Katz [1]†, Rachel Zaguri[1]†, Simon Edvardson[2]†, Channa Maayan[2], Orly Elpeleg[3], Shaya Lev[1], Elyad Davidson[4], Maximilian Peters[1], Shlomit Kfir-Erenfeld[5], Esther Berger[6], Shifa Ghazalin[1], Alexander M. Binshtok[1]* and Baruch Minke[1]*

*Corresponding authors: Email: baruch.minke@mail.huji.ac.il (B.M.);
alexander.binshtok@mail.huji.ac.il (A.M.B.)

This PDF file includes:

Supplementary Methods

Supplemental Figures1 to 9

Supplemental Tables 1 to 2

Supplementary Methods:

RNA isolation from Human PBMC:

Peripheral blood (10 mL), obtained from 5 donors (3 men and 2 women) and from A1 were collected in sterile heparin tubes (6-455084, De-Groot). The blood was diluted with PBS (1:1) and the PBMCs were separated by centrifugation over Histopaque (10771, Sigma-Aldrich) at 400*g* for 30 minutes at 20°C. The mononuclear layer, consisting predominantly of lymphocytes, was collected and centrifuged at 160*g* for 10 minutes at 20°C. The pellet was resuspended in 10 mL of PBS and then centrifuged at 160*g* for 10 minutes at 20°C. The pellet was finally resuspended in 1 mL TriReagent (00901023310, Bio-Lab) and RNA was extracted according to the manufacturer's instructions. The concentration of RNA was measured, and samples were stored at −20°C until use.

Quantification of gene expression by real-time reverse transcription:

1 μg of RNA (pretreated with DNase I kit (AMPD1, Sigma)) was used to generate cDNA using the High-Capacity cDNA reverse-transcription Kit containing RNase inhibitor (AB-4374966, Thermo-Fisher scientific). A TaqMan Gene Expression Assay (Applied Biosystems) for *TRPV1* (Hs00218912_m1) was used to measure mRNA expression levels relative to the endogenous reference gene, *GAPDH* (4333764F). Measurements were performed using StepOnePlus Real-Time PCR (4376600, Thermo-Fisher scientific). Each reaction was performed in triplicate and was accompanied by a negative control in which the RNA was replaced by nuclease-free water. Comparative threshold cycle (C_T) was used to calculate differential mRNA. ΔC_T values were calculated as the difference between the mean C_T values for *TRPV1* and the mean C_T values for *GAPDH* ($\overline{C_{T,TRPV1}}$ - $\overline{C_{T,GAPDH}}$). $\Delta\Delta C_T$ values were calculated as the difference between ΔC_T values and the mean ΔC_T value of healthy volunteers. Data are presented as fold change ($2^{-\Delta\Delta C_T}$).

TRPV1 Exome sequencing:

Genomic DNA was extracted from the blood using VERSAGENETM DNA Purification Kit (Gentra, Minneapolis, MN). The *TRPV1* genomic DNA was fragmented into several pieces which overlapped partially with the contiguous regions by PCR primers. Sequences of the DNA fragments were assembled into the whole *TRPV1* gene, after sequencing. First strand cDNA was synthesized with Superscript^{TM} (Invitrogen). The genomic DNA or cDNA fragments were amplified by PCR using EF-Taq DNA polymerase (SolGent, Daejeon, South Korea). The PCR product which was purified with the QIAquick PCR purification kit (QIAGEN, Valencia, CA) was cloned into TOPO TA vector (Invitrogen). Single-pass sequencing was performed in an ABI 3730xl sequencer (Applied Biosystems), following the manufacturers' protocols.

Exome Analysis:

Exonic sequences from DNA samples of the index case were enriched with the SureSelect Human All Exon 50 Mb V.5 Kit (Agilent Technologies, Santa Clara, California, USA). Sequences (125-bp paired-end) were generated on a HiSeq2500 (Illumina, San Diego, California, USA). Read alignment and variant calling were performed with DNAnexus (Palo Alto, California, USA) using default parameters with the human genome assembly hg19 (GRCh37) as reference. Parental consent was given for genetic studies. The study was performed with the approval of the ethical committees of Hadassah Medical Center and the Israeli Ministry of Health.

Whole exome sequencing (WES) and exome analysis:

WES of A1 was performed as follows: Exonic sequences from DNA sample of the index case were enriched with the SureSelect Human All Exon 50 Mb V.5 Kit (Agilent Technologies,

Santa Clara, California, USA). Sequences (125-bp paired-end) were generated on a HiSeq2500 (Illumina, San Diego, California, USA). Read alignment and variant calling were performed with DNAnexus (Palo Alto, California, USA) using default parameters with the human genome assembly hg19 (GRCh37) as reference. The analysis yielded 64.1 million mapped reads with a mean coverage of 114X. Following alignment and variant calling, a series of filtering steps was performed. These included removing variants which were called less than 8X, were off-target, heterozygous, synonymous, had minor allele frequency (MAF)>0.5% at ExAC or MAF>1% at the Hadassah in-house database (approximately 900 ethnic-matched exome sequences).

WES of A2, E13 and E14 was performed as follows: exonic sequences was performed as recently described (PMID: 34514401). The analysis yielded 56.9, 75 and 89 million mapped reads, with a mean coverage of 90X, 116X and 137X and 94.7%, 95.03% and 95.3% over 20X, respectively. Following alignment and variant calling, a series of filtering steps was performed. These included removing variants which were exonic and splice-site, non-synonymous variants using GERP>2.0, heterozygous and had MAF>0.2% at GnomAD or MAF>1% at the Hadassah in-house database (comprised of 11,000 WES). The study was performed with the approval of the ethical committees of Hadassah Medical Center and the Israeli Ministry of Health.

Taste experiment:

Square 10x10 mm gauze pads were soaked in solution containing a tastant or spices, each at 5 different concentrations (except for capsaicin which was given at an additional higher concentration to the affected individuals). The pads were moved on the anterior part of the tongue and mouth. The tastants were applied in ascending order and minimum recognition threshold was measured. Sodium chloride (793566), sucrose (S0839), citric acid (C0759),

quinine hydrochloride (822194), cinnamaldehyde (W228613), menthol (M2780), capsaicin (M2028). All chemicals were purchased from Sigma-Aldrich.

The taste experiments were performed with prior knowledge of the genotype.

Histamine test:

A volume of 0.05 mL of histamine bisphosphate monohydrate (H7375, Sigma-Aldrich) dissolved in DDW at 1:1000 w/v and 1:10,000 w/v was injected intracutaneously into the forearm of A1 using a needle (25G, BD Microlance). DDW was used as vehicle. Images were processed for the extent of the flare using an imaging tool (Magic wand, tolerance 5, Adobe Photoshop) for detecting areas of similar tone and color.

Skin biopsy and immunohistochemistry:

Skin sample was collected from the bottom part of the leg adjacent to the ankle using a skin biopsy Punch 3 mm x 10 (Integra industries, NY, USA) after anesthetizing the area using Esracain injection 1% (Lidocaine Hydrochloride 10 mg/mL solution, Rafa industries LTD, Jerusalem, Israel). The skin biopsy was put into 4% paraformaldehyde in PBS for 24 hours before further processed. Immunohistochemistry was performed on deparaffinized 4 μm sections of paraffin-embedded tissue blocks. The detection was done using an anti-PGP9.5 antibody (Z5116, Dako Denmark) diluted to a titer of 1:150, and Ultra View DAB Detection Kit, running on an automated system (Ventana BenchMark Ultra Autostainer, Tucson, AZ, USA). 3,3'-Diaminobenzidin (DAB) was used as chromogen. All sections were counterstained with Mayers hematoxylin. Images were taken using CCD digital camera (ORCA-ER, Hamamatsu) controlled by SimplePCI software (Hamamatsu, Version 6.1) mounted on an Axioskop (Zeiss) microscope with x20/0.45 achrostigmat (Zeiss) objective.

Medical inquiry:

All medical inquiries were performed by a physician in the presence of a hebrew-arabic translator with a medical background. The medical inquiry of the children was performed in the presence of the parents and answers were given by the children and their parents. For the affected individual A2, the mother of the child answered the medical inquiry and therefore some of her answers were considered as “impressions”.

Sequence alignment:

We performed the multiple sequence alignment using Geneious Alignment within the Geneious Prime (2020.2.2) software using the default parameters (cost matrix: Blosum62) in Global alignment mode. Consensus sequence threshold was set to 100%-identity. Numbering is according to hTRPV1.

Software:

Zen 3.5 (blue edition, Zeiss); Geneious Prime (2020.2.2); Clampex (10.7.0.3) and Clampfit (11.1.0.23, Molecular Devices); NIS-elements Ar (4.50, Nikon), OriginPro 2017 (64bit, SR2 b9.4.2.380, OriginLab), CorelDRAW and CorelPHOTO-PAINT 2018 (20.0.0.633), Photoshop CC (20.0.7, Adobe), Prism 8 for macOS (8.4.0), IBM SPSS Statistics (26.0.0.1), Word and Excel 2019 MSO (16.0.10363.20015, Microsoft) 64-bit.

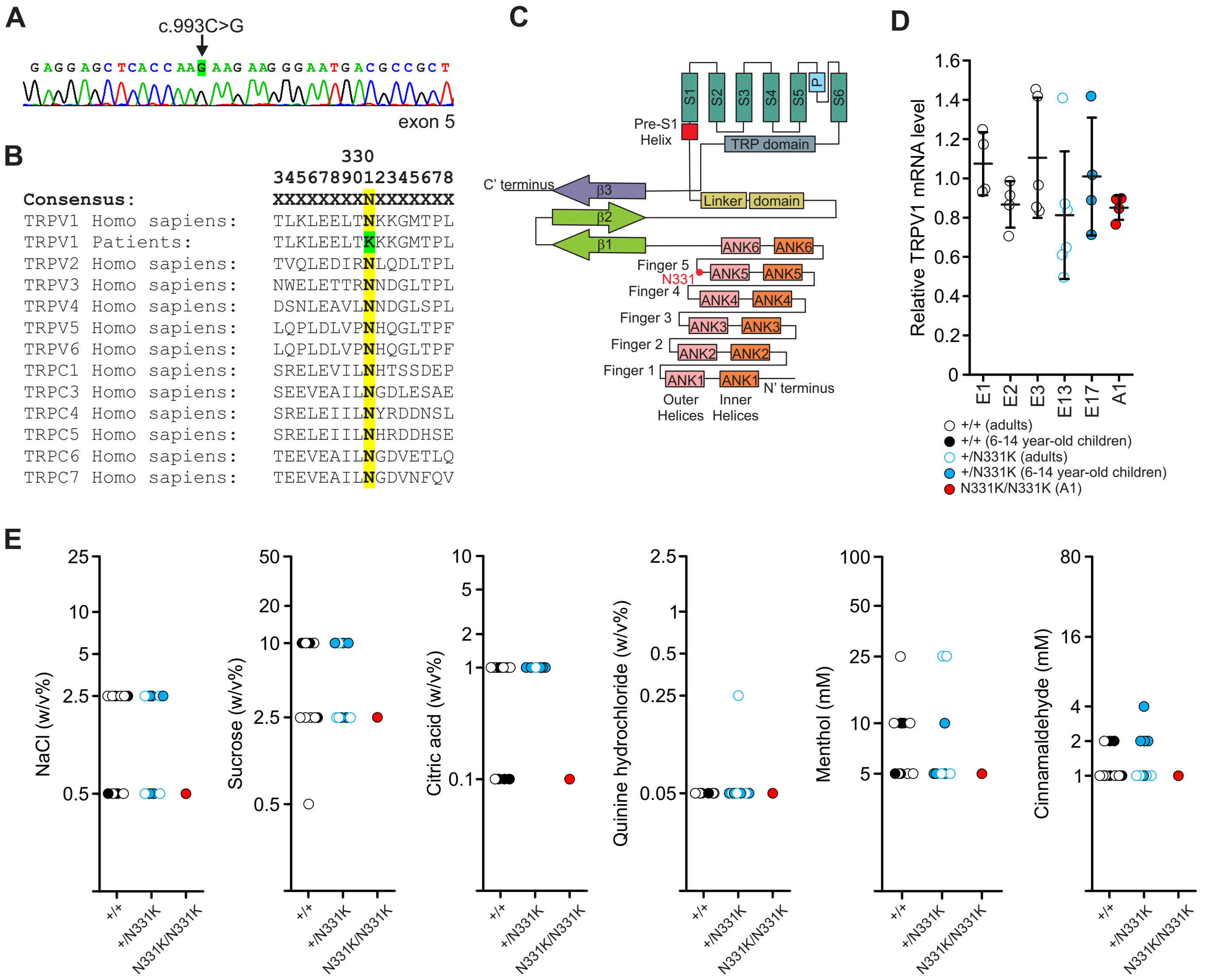
A
c.993C>G
GAGGAGCTCACCAAGAAGAAGGGAATGACGCCGCT
exon 5
B
330
345678901234567 8
Consensus: XXXXXXXXNXXXXXXX
TRPV1 Homo sapiens: TLKLEELTNKKGMTPL
TRPV1 Patients: TLKLEELTKKKGMTPL
TRPV2 Homo sapiens: TVQLEDIRNLQDLTPL
TRPV3 Homo sapiens: NWELETTRNNDGLTPL
TRPV4 Homo sapiens: DSNLEAVLNNDGLSPL
TRPV5 Homo sapiens: LQPLDLVPNHQGLTPF
TRPV6 Homo sapiens: LQPLDLVPNHQGLTPF
TRPC1 Homo sapiens: SRELEVILNHTSSDEP
TRPC3 Homo sapiens: SEEVEAILNGDLESAE
TRPC4 Homo sapiens: SRELEIILNYRDDNSL
TRPC5 Homo sapiens: SRELEIILNHRDDHSE
TRPC6 Homo sapiens: TEEVEAILNGDVETLQ
TRPC7 Homo sapiens: TEEVEAILNGDVNFQV
C
S1 S2 S3 S4 S5 P S6
Pre-S1 Helix
TRP domain
C' terminus
β3 β2 β1
Linker domain
ANK6 ANK6
Finger 5
N331
ANK5 ANK5
Finger 4
ANK4 ANK4
Finger 3
ANK3 ANK3
Finger 2
ANK2 ANK2
Finger 1
ANK1 ANK1
N' terminus
Outer Helices
Inner Helices
D
Relative TRPV1 mRNA level
0 0.2 0.4 0.6 0.8 1.0 1.2 1.4 1.6
E1 E2 E3 E13 E17 A1
+/+ (adults)
+/+ (6-14 year-old children)
+/N331K (adults)
+/N331K (6-14 year-old children)
N331K/N331K (A1)
E
NaCl (w/v%)
0.5 2.5 5 10 25
Sucrose (w/v%)
0.5 2.5 10 20 50
Citric acid (w/v%)
0.1 1 2 5 10
Quinine hydrochloride (w/v%)
0.05 0.25 0.5 1 2.5
Menthol (mM)
5 10 25 50 100
Cinnamaldehyde (mM)
1 2 4 16 80
+/+
+/N331K
N331K/N331K

Supplemental Figure 1. Expression levels of TRPV1 mRNA and recognition threshold of basic tastants. (**A**) Exon sequencing of the *TRPV1* gene of A1 (shown) and A2 revealed a single nucleotide substitution, C993G in exon 5 of the *TRPV1* gene. (**B**) Sequence alignment showing the conservation of asparagine (N, yellow) residue in all channel members of the human TRPV and TRPC subfamilies. (**C**) A scheme of the protein domains of TRPV1 showing the location of the missense mutation. Ankyrin-repeat domains (ANK); transmembrane domain (S); Pore domain (P). (**D**) Relative *TRPV1* mRNA levels obtained from PBMCs of healthy volunteers (black, $n_{+/+}^{E1} = 4$, $n_{+/+}^{E2} = 4$, $n_{+/+}^{E3} = 5$), heterozygote family members (cyan, $n_{+/N331K}^{E13} = 6$, $n_{+/N331K}^{E17} = 4$) and A1 (red, $n_{N331K/N331K} = 4$ repeats) were determined by quantitative real time PCR (error bars indicate SD). (**E**) Recognition thresholds to NaCl (responded to the compound as “salty”), sucrose (responded to the compound as “sweet”), citric acid (responded to the compound as “sour”), quinine hydrochloride (responded to the compound as “bitter”), menthol (TRPM8 activator, responded to the compound as “mint”) and cinnamaldehyde (TRPA1 activator, responded to the compound as “cinnamon”, $n_{+/+} = 13$, $n_{+/N331K} = 9$, $n_{N331K/N331K}$ = minimum of 3 repeats).

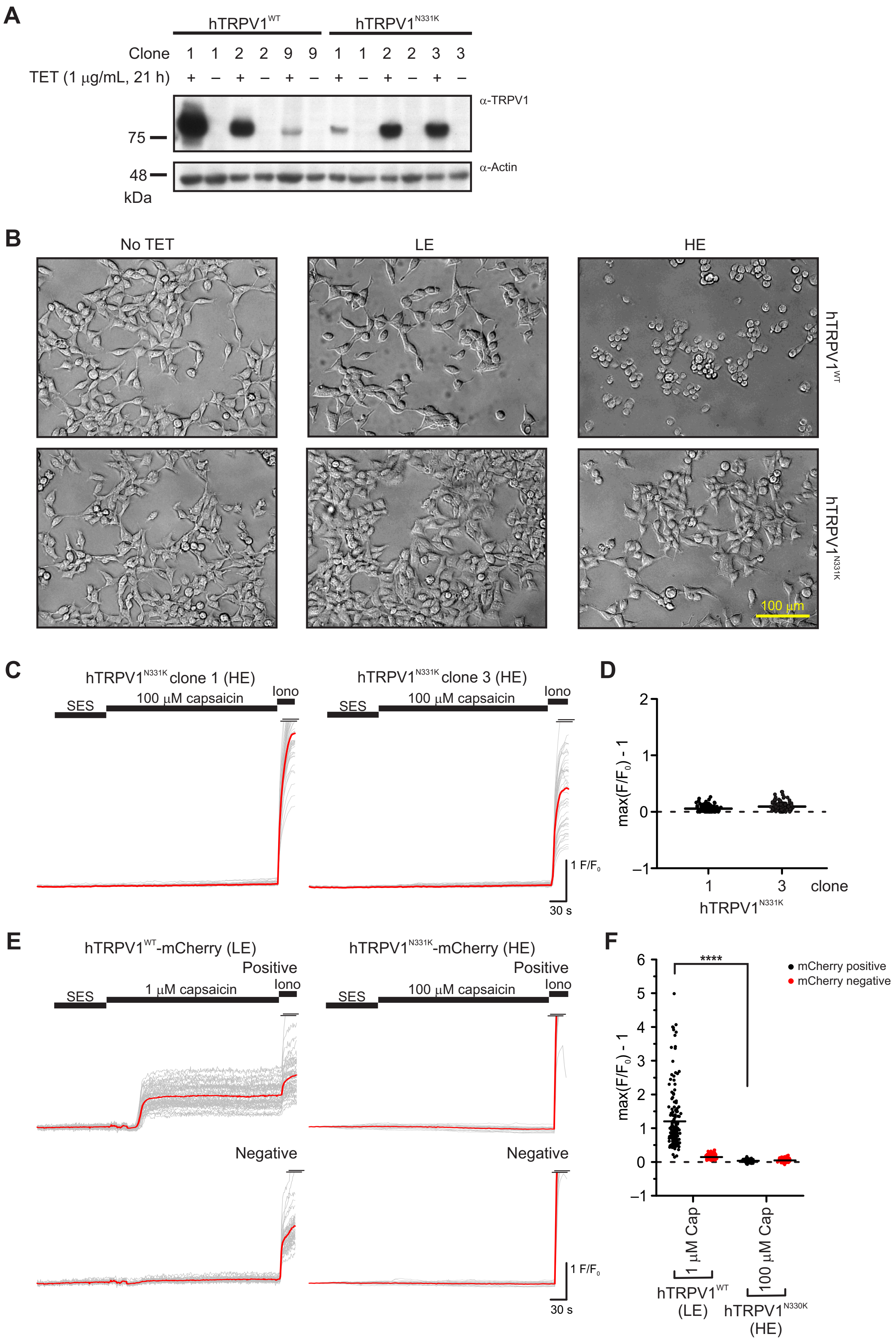
A
hTRPV1WT
hTRPV1N331K
Clone 1 1 2 2 9 9 1 1 2 2 3 3
TET (1 μg/mL, 21 h) + – + – + – + – + – + –
α-TRPV1
75
α-Actin
48
kDa
B
No TET
LE
HE
hTRPV1WT
hTRPV1N331K
100 μm
C
hTRPV1N331K clone 1 (HE)
hTRPV1N331K clone 3 (HE)
SES
100 μM capsaicin
Iono
1 F/F0
30 s
D
max(F/F0) - 1
clone
hTRPV1N331K
E
hTRPV1WT-mCherry (LE)
hTRPV1N331K-mCherry (HE)
Positive
Negative
1 μM capsaicin
100 μM capsaicin
F
****
mCherry positive
mCherry negative
1 μM Cap
100 μM Cap
hTRPV1WT (LE)
hTRPV1N330K (HE)

Supplemental Figure 2. hTRPV1^{N331K} stably and transiently expressed in T-REx-293 cell line is nonfunctional. (**A**) Western-blot analysis of T-REx-293 cells stably expressing different clones of hTRPV1^{WT} or hTRPV1^{N331K} using an anti-TRPV1 antibody. Anti-actin antibody was used as protein loading control (*n* = 3). Clones 2 of T-REx-293 cells stably expressing hTRPV1^{WT} or hTRPV1^{N331K} were selected for the experiments of Figures 2, 3, 6C, 7 and Supplemental Figure 3. (**B**) Photographs of T-REx-293 cells stably expressing hTRPV1^{WT} or hTRPV1^{N331K} at various doses and duration of TET incubation. Note that T-REx-293 cells stably expressing hTRPV1^{WT} show major change in morphology (round shape, right) after incubation with 1 μg/mL TET for 21 hours, which was not observed in T-REx-293 cells stably expressing hTRPV1^{N331K} (scale bar:100 μm, the scale bar is applicable to all panels). (**C**) Representative traces of Fura-2 based Ca^{2+} imaging from T-REx-293 cell lines stably expressing hTRPV1^{N331K} clone 1 (left) or hTRPV1^{N331K} clone 3 (right) in response to application of 100 μM capsaicin. Application of the Ca^{2+} ionophore ionomycin (Iono) served as a positive control. (**D**) Maximal (max) normalized fluorescence changes from baseline during application of 100 μM capsaicin (Cap): hTRPV1^{N331K} HE, clone 1, 100 μM Cap (*N* = 4 plates, *n* = 165 cells) and hTRPV1^{N331K} HE, clone 3, 100 μM Cap (*N* = 4, *n* = 157). (**E**) Representative traces of Fura-2 based Ca^{2+} imaging from T-REx-293 cell lines transiently transfected with hTRPV1^{WT}-mCherry at LE levels (left) and hTRPV1^{N331K}-mCherry at HE levels (right) in response to application of 1 μM or 100 μM capsaicin, respectively. Upper traces are mCherry positive cells (Positive) and lower traces are mCherry negative cells (Negative). Application of the Ca^{2+} ionophore ionomycin served as a positive control. (**F**) Maximal (max) normalized fluorescence change from baseline during application of 1 μM capsaicin: hTRPV1^{WT} LE, mCherry positive, 1 μM Cap (*N* = 6, *n* = 169), hTRPV1^{WT} LE, mCherry negative, 1 μM Cap (*N* = 6, *n* = 227) and 100 μM capsaicin: hTRPV1^{N331K} HE, mCherry positive, 100 μM Cap (*N* = 4, *n* = 102), hTRPV1^{N331K} HE, mCherry negative, 100 μM Cap (*N* = 4, *n* = 124). ****P < 0.0001 by 2-tailed Student's *t*-test.

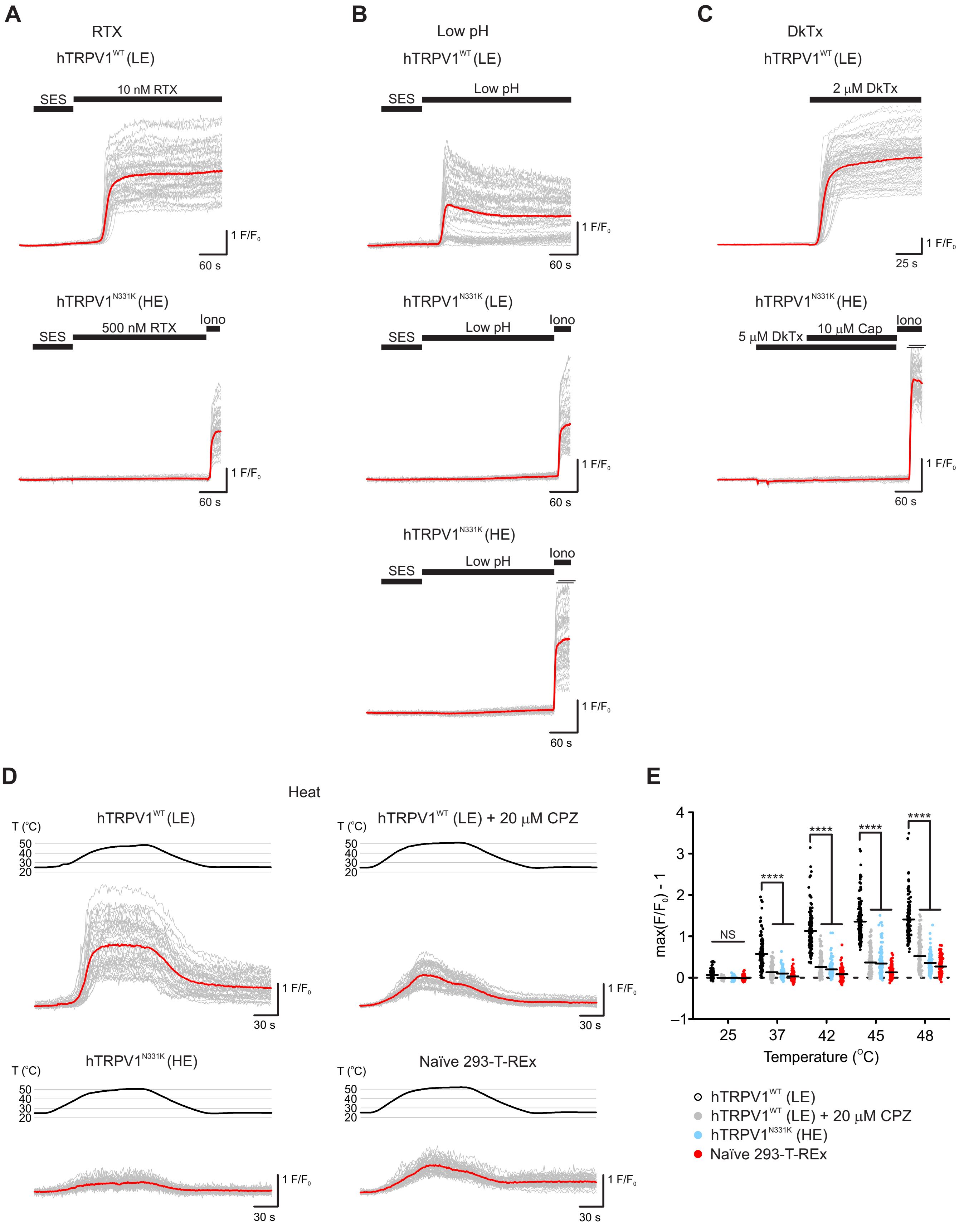
A
RTX
hTRPV1WT (LE)
SES
10 nM RTX
1 F/F0
60 s
hTRPV1N331K (HE)
SES
500 nM RTX
Iono
1 F/F0
60 s
B
Low pH
hTRPV1WT (LE)
SES
Low pH
1 F/F0
60 s
hTRPV1N331K (LE)
SES
Low pH
Iono
1 F/F0
60 s
hTRPV1N331K (HE)
SES
Low pH
Iono
1 F/F0
60 s
C
DkTx
hTRPV1WT (LE)
2 μM DkTx
1 F/F0
25 s
hTRPV1N331K (HE)
5 μM DkTx
10 μM Cap
Iono
1 F/F0
60 s
D
Heat
T (°C)
hTRPV1WT (LE)
50
40
30
20
1 F/F0
30 s
hTRPV1WT (LE) + 20 μM CPZ
hTRPV1N331K (HE)
Naïve 293-T-REx
E
max(F/F0) - 1
4
3
2
1
0
−1
NS
****
25
37
42
45
48
Temperature (°C)
hTRPV1WT (LE)
hTRPV1WT (LE) + 20 μM CPZ
hTRPV1N331K (HE)
Naïve 293-T-REx

Supplemental Figure 3. The N331K mutation renders the TRPV1 channel nonfunctional. (A-D) Representative traces of Fura-2 based Ca^{2+} imaging from T-REx-293 cell lines stably expressing $hTRPV1^{WT}$ at LE levels, $hTRPV1^{N331K}$ at LE or HE levels in response to various TRPV1 agonists. **(A)** $hTRPV1^{WT}$, LE, 10 nM RTX (top), $hTRPV1^{N331K}$, HE, 500 nM RTX (bottom); (**B)** $hTRPV1^{WT}$ LE, low pH (top), $hTRPV1^{N331K}$ LE, low pH (middle), $hTRPV1^{N331K}$ HE, low pH (bottom); **(C)** $hTRPV1^{WT}$ LE, 2 μM DkTx (top), $hTRPV1^{N331K}$ HE, 5 μM DkTx plus 10 μM Cap (bottom); **(D)** $hTRPV1^{WT}$ LE, heat (top left), $hTRPV1^{WT}$ LE incubated with 20 μM Capsazepine (CPZ), heat (top right), $hTRPV1^{N331K}$ HE, heat (bottom left), naïve T-Rex-293, heat (bottom right). Application of the Ca^{2+} ionophore ionomycin (Iono) served as a positive control. **(E)** Maximal (max) normalized fluorescence change from baseline during heat ramp from 25 to 50°C at various temperatures: $hTRPV1^{WT}$ LE, heat (N = 4 plates, n = 155 cells), $hTRPV1^{WT}$ LE incubated with 20 μM CPZ, heat (N = 3, n = 155), $hTRPV1^{N331K}$ HE, heat (N = 6, n = 155), Naïve T-REx-293, heat (N = 6, n = 155). ****$P<0.0001$ and NS – non-significant $P>0.05$, by 2-way ANOVA with Bonferroni's multiple comparisons test.

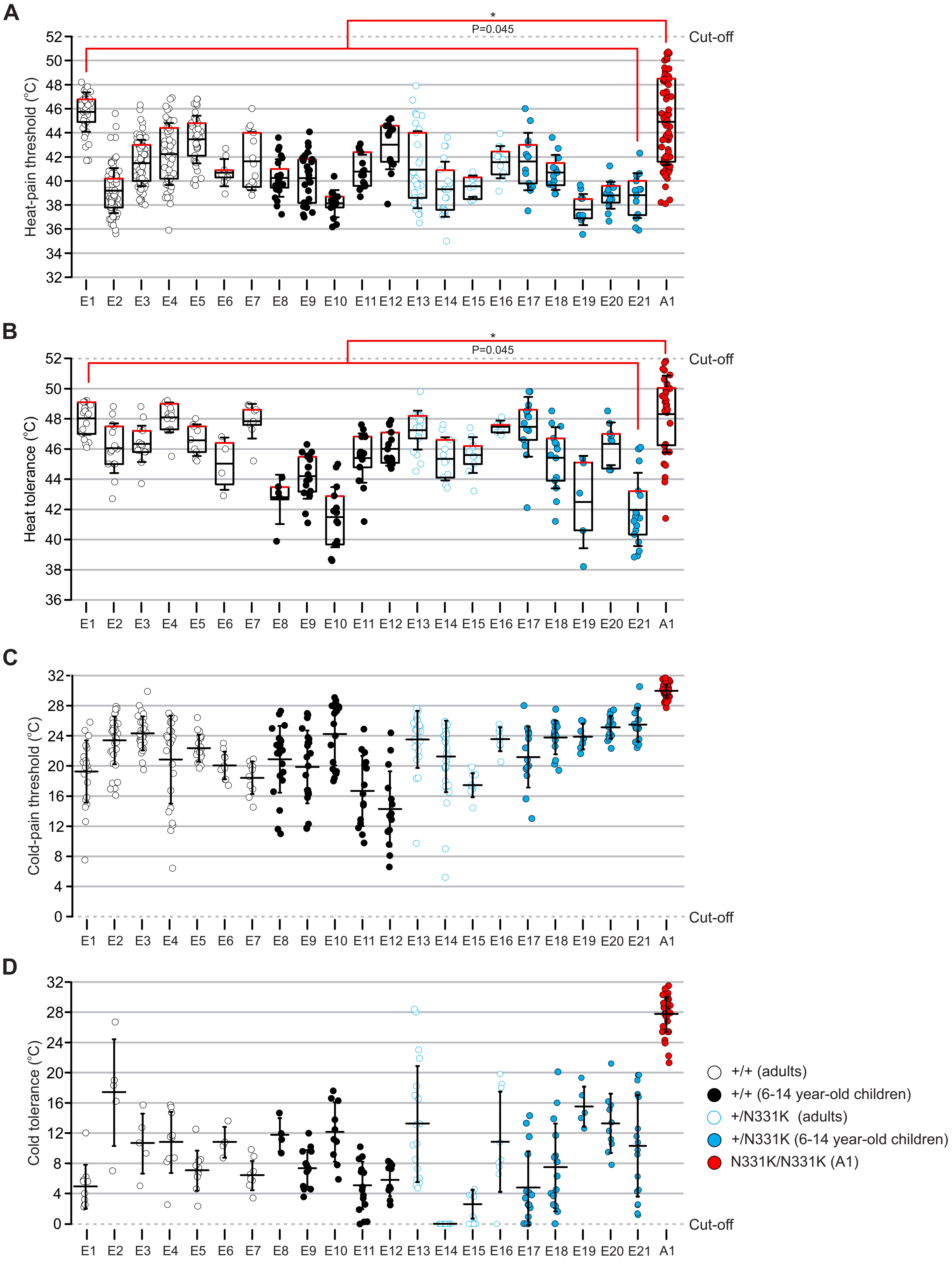

A
B
C
D
Heat-pain threshold (°C)
Heat tolerance (°C)
Cold-pain threshold (°C)
Cold tolerance (°C)
*
P=0.045
Cut-off
E1 E2 E3 E4 E5 E6 E7 E8 E9 E10 E11 E12 E13 E14 E15 E16 E17 E18 E19 E20 E21 A1
+/+ (adults)
+/+ (6-14 year-old children)
+/N331K (adults)
+/N331K (6-14 year-old children)
N331K/N331K (A1)

Supplemental Figure 4: The Affected Individual (A1) demonstrates elevated heat-pain threshold and elevated cold-pain threshold compared with control groups. (**A-D**) Repeated measurements of **(A)** HPT **(**n^{E1} = 37, n^{E2} = 93, n^{E3} = 73, n^{E4} = 53, n^{E5} = 38, n^{E6} = 9, n^{E7} = 14, n^{E8} = 24, n^{E9} = 25, n^{E10} = 15, n^{E11} = 15, n^{E12} = 15, n^{E13} = 30, n^{E14} = 19, n^{E15} = 10, n^{E16} = 12, n^{E17} = 13, n^{E18} = 12, n^{E19} = 10, n^{E20} = 15, n^{E21} = 13, n^{A1} = 74), (**B**) HT (n^{E1} = 19, n^{E2} = 15, n^{E3} = 15, n^{E4} = 15, n^{E5} = 9, n^{E6} = 4, n^{E7} = 9, n^{E8} = 5, n^{E9} = 17, n^{E10} = 15, n^{E11} = 15, n^{E12} = 15, n^{E13} = 19, n^{E14} = 11, n^{E15} = 10, n^{E16} = 9, n^{E17} = 14, n^{E18} = 15, n^{E19} = 5, n^{E20} = 9, n^{E21} = 18, n^{A1} = 32), (**C**) CPT (n^{E1} = 24, n^{E2} = 36, n^{E3} = 30, n^{E4} = 25, n^{E5} = 19, n^{E6} = 10, n^{E7} = 10, n^{E8} = 22, n^{E9} = 25, n^{E10} = 20, n^{E11} = 15, n^{E12} = 15, n^{E13} = 25, n^{E14} = 35, n^{E15} = 9, n^{E16} = 8, n^{E17} = 13, n^{E18} = 15, n^{E19} = 7, n^{E20} = 15, n^{E21} = 14, n^{A1} = 53) and (**D**) CT (n^{E1} = 10, n^{E2} = 5, n^{E3} = 5, n^{E4} = 10, n^{E5} = 10, n^{E6} = 4, n^{E7} = 9, n^{E8} = 4, n^{E9} = 14, n^{E10} = 10, n^{E11} = 15, n^{E12} = 10, n^{E13} = 20, n^{E14} = 15, n^{E15} = 10, n^{E16} = 9, n^{E17} = 15, n^{E18} = 15, n^{E19} = 5, n^{E20} = 10, n^{E21} = 18, n^{A1} = 35) from healthy volunteers (E1-E12), heterozygote family members (E13-E21) and A1. Note the scatter of the individually measured points of high temperature measurements in A1, when testing for HPT and HT, which are not observed in individuals of the control groups. (Box plot; top red lines indicate upper 75% quartile; the middle line indicates mean; error bars indicate SD). We therefore decided to compare the upper quartile of HPT and HT measurements of A1 with those of the control groups and found that it is significantly greater. However, it should be borne in mind, that this is a data-driven hypothesis and, as such, is to be interpreted with caution. P=0.045, by 1-tailed Mann-Whitney *U*-test.

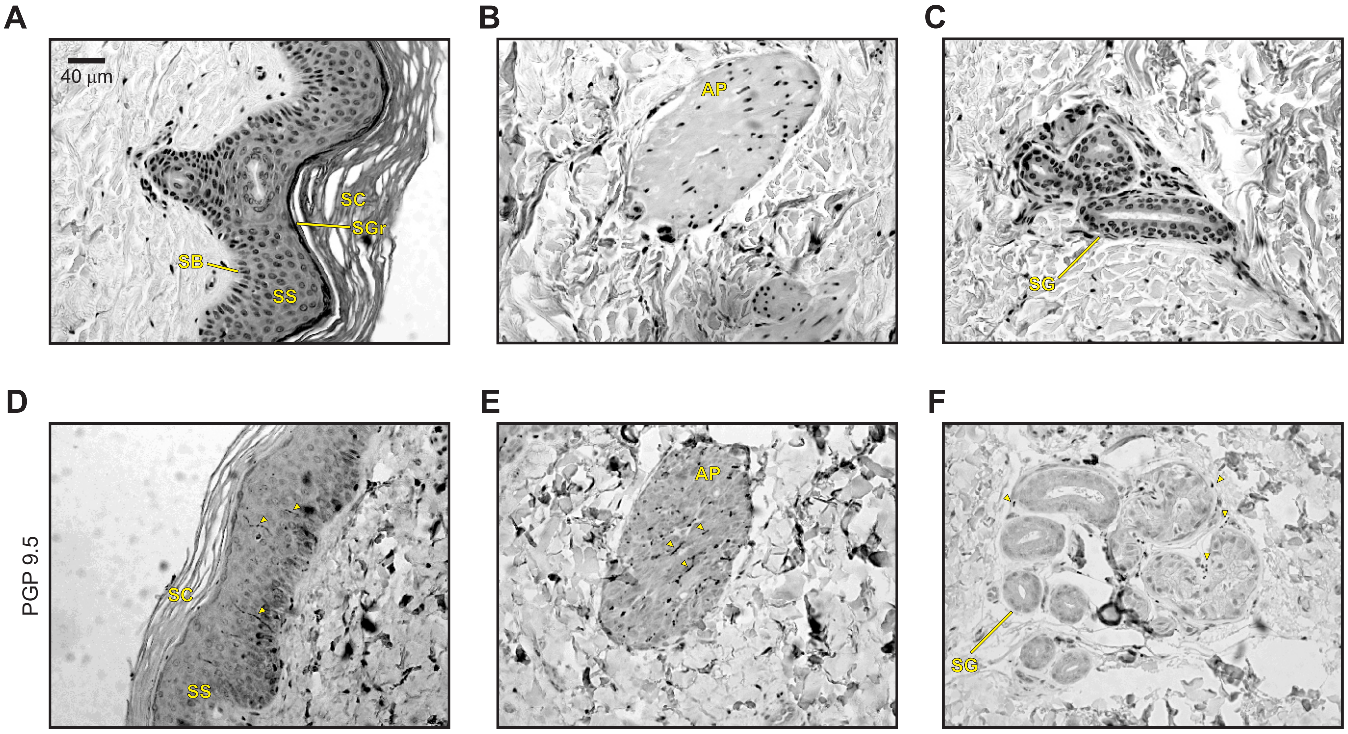
A
40 µm
SC
SGr
SB
SS
B
AP
C
SG
D
PGP 9.5
SC
SS
E
AP
F
SG

Supplemental Figure 5: Skin biopsy of A1 shows intact small nerve fibers and normal skin structures. Photomicrograph of thick skin section taken from a skin biopsy of A1's ankle. (**A-C**) hematoxylin staining. (**D-F**) Immuno staining using PGP9.5 antibody. **SC** - Stratum Corneum, **SS** - Stratum Spinosum, **SGr** - Stratum Granulosum, **SB** - Stratum Basale, **AP** – Arrector Pilus, **SG** - Sweat Gland. Scale bar: 40 μm (the scale bar is applicable to all panels).

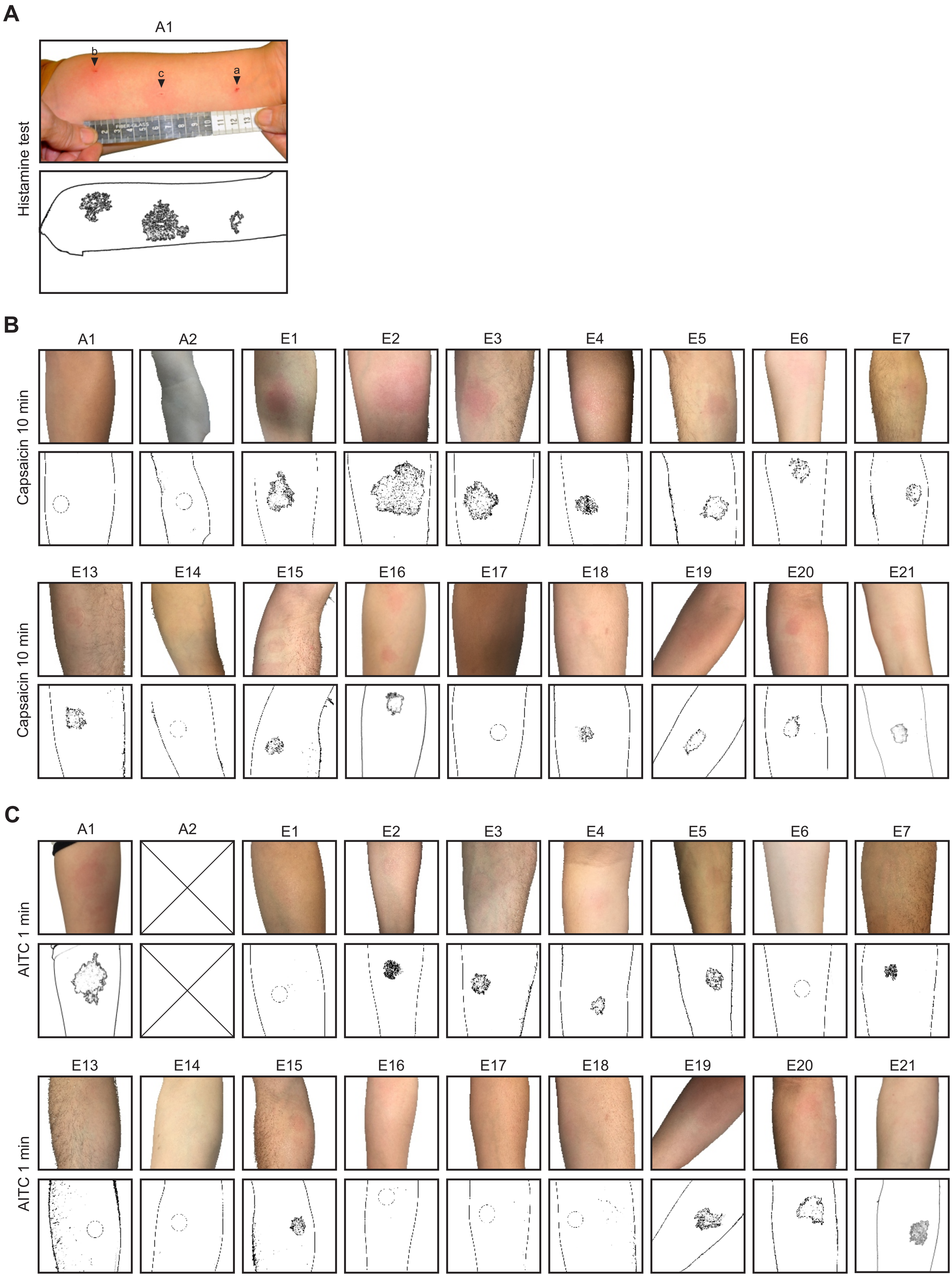
A
A1
b
c
a
Histamine test
B
A1
A2
E1
E2
E3
E4
E5
E6
E7
Capsaicin 10 min
E13
E14
E15
E16
E17
E18
E19
E20
E21
Capsaicin 10 min
C
A1
A2
E1
E2
E3
E4
E5
E6
E7
AITC 1 min
E13
E14
E15
E16
E17
E18
E19
E20
E21
AITC 1 min

Supplemental Figure 6: Histamine and AITC but not capsaicin induces flare responses in A1. (**A**) Image of the hand of A1 after 3 injections (top): vehicle DDW (**a**), 1:10,000 w/v histamine (**b**) 1:1,000 w/v histamine (**c**). Images were processed (bottom) for the extent of the flare using an imaging tool for detecting areas of similar tone and color. (**B**) Images of individual forearms (top) from healthy volunteers (E1-E7), heterozygote family members (E13-E21) and the affected individuals (A1 and A2) after topical application of 5% capsaicin for 10 minutes using Finn chamber. Images were processed (bottom) for the extent of the flare using an imaging tool for detecting areas of similar tone and color. Note that the two affected individuals did not develop any flare, while most of the individuals in the control groups developed skin flare (14/16). (**C**) Images of individual forearms after topical application of 25% AITC for 1 minute. Note that A1 developed a large skin flare, while most individuals in the control groups developed a small flare (4/16) or did not develop any flare (7/16).

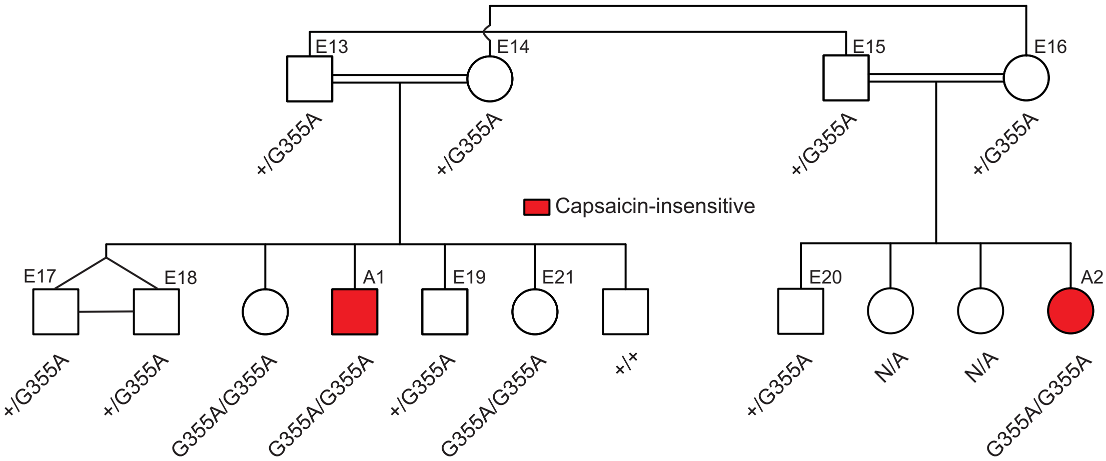
E13
E14
E15
E16
+/G355A
+/G355A
+/G355A
+/G355A
Capsaicin-insensitive
E17
E18
A1
E19
E21
E20
A2
+/G355A
+/G355A
G355A/G355A
G355A/G355A
+/G355A
G355A/G355A
+/+
+/G355A
N/A
N/A
G355A/G355A

Supplemental Figure 7: Genetic analysis of PROKR1 mutation in the extended family.

Pedigree chart of a missense mutation in the *PROKR1* gene in the extended family. 4 family members were found to carry a homozygous *PROKR1* c.355G>A mutation. 2 of them also carry the homozygous *TRPV1* c.993C>G mutation (A1 and A2), while the others are heterozygote to the *TRPV1* c.993C>G mutation (one of them is E21).

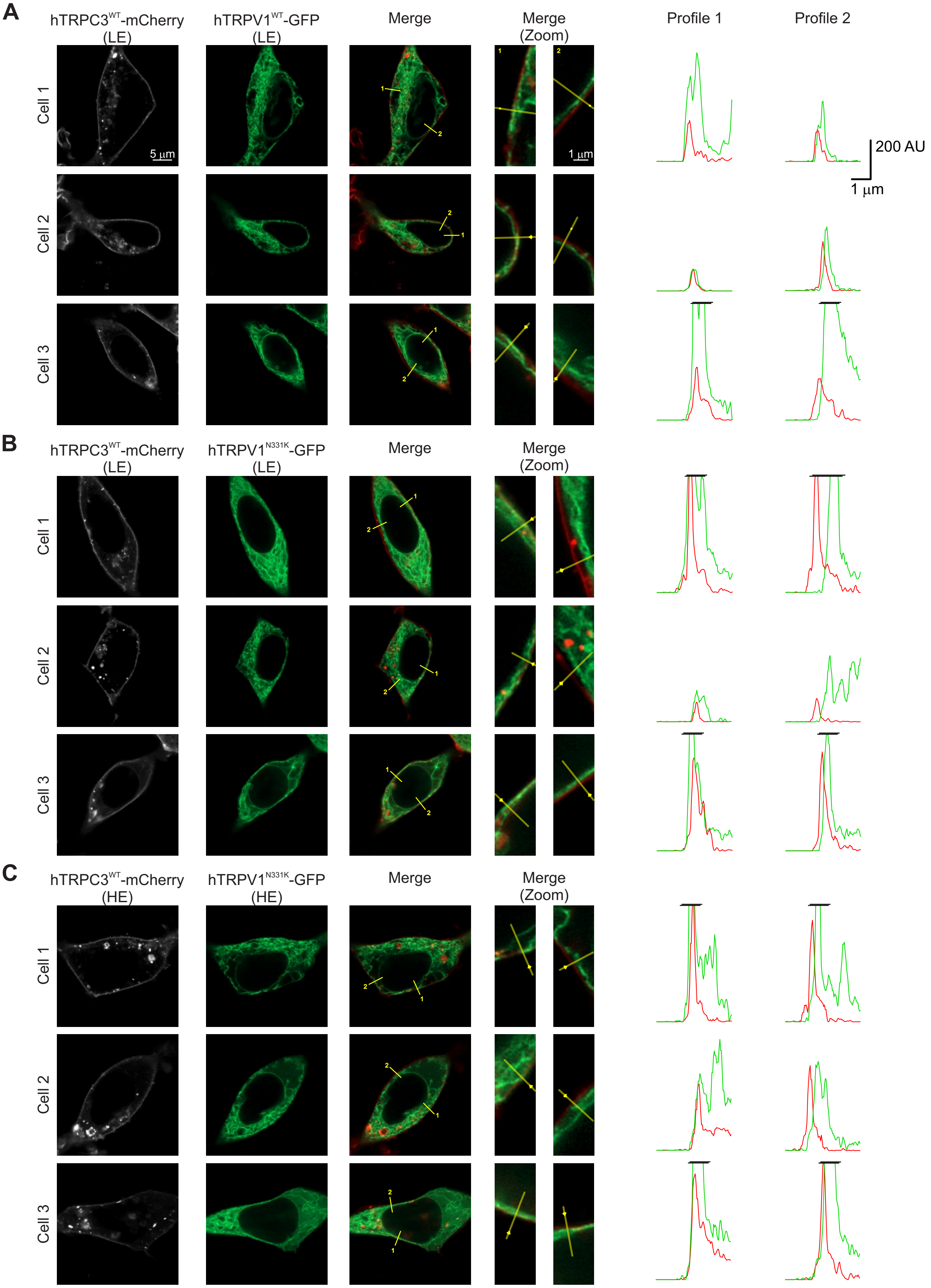

A
hTRPC3WT-mCherry (LE)
hTRPV1WT-GFP (LE)
Merge
Merge (Zoom)
Profile 1
Profile 2
Cell 1
Cell 2
Cell 3
5 µm
1 µm
200 AU
1 µm
B
hTRPC3WT-mCherry (LE)
hTRPV1N331K-GFP (LE)
Merge
Merge (Zoom)
C
hTRPC3WT-mCherry (HE)
hTRPV1N331K-GFP (HE)
Merge
Merge (Zoom)

Supplemental Figure 8: hTRPV1^{N331K} channel reach the plasma membrane.

Representative confocal images of naïve T-REx-293 cells co-transfected with hTRPC3^{WT}-mCherry (red or white) together with hTRPV1^{WT}-GFP or hTRPV1^{N331K}-GFP (green). First column (from left to right): Confocal images of hTRPC3^{WT}-mCherry (white). Scale bar: 5 μm (the scale bar is applicable to the first, second and third columns). Second column: Confocal images of hTRPV1^{WT}-GFP (green) at LE levels (**A**) and hTRPV1^{N331K}-GFP (green) at LE levels (**B**) and at HE levels (**C**). Third column: Confocal merged images showing hTRPV1-GFP (green) and hTRPC3^{WT}-mCherry (red). Fourth and Fifth columns: High magnification confocal merged images showing hTRPV1-GFP (green) and hTRPC3^{WT}-mCherry (red). Scale bar: 1 μm (the scale bar is applicable to the fourth and fifth columns). Profile graphs indicate the fluorescence intensity along the yellow lines crossing the plasma membrane in the merged images (third, fourth and fifth columns). Note that in profile 1 the GFP fluorescent signal precedes or coincides with that of the mCherry, while in profile 2 the mCherry fluorescent signal precedes or coincides with that of the GFP.

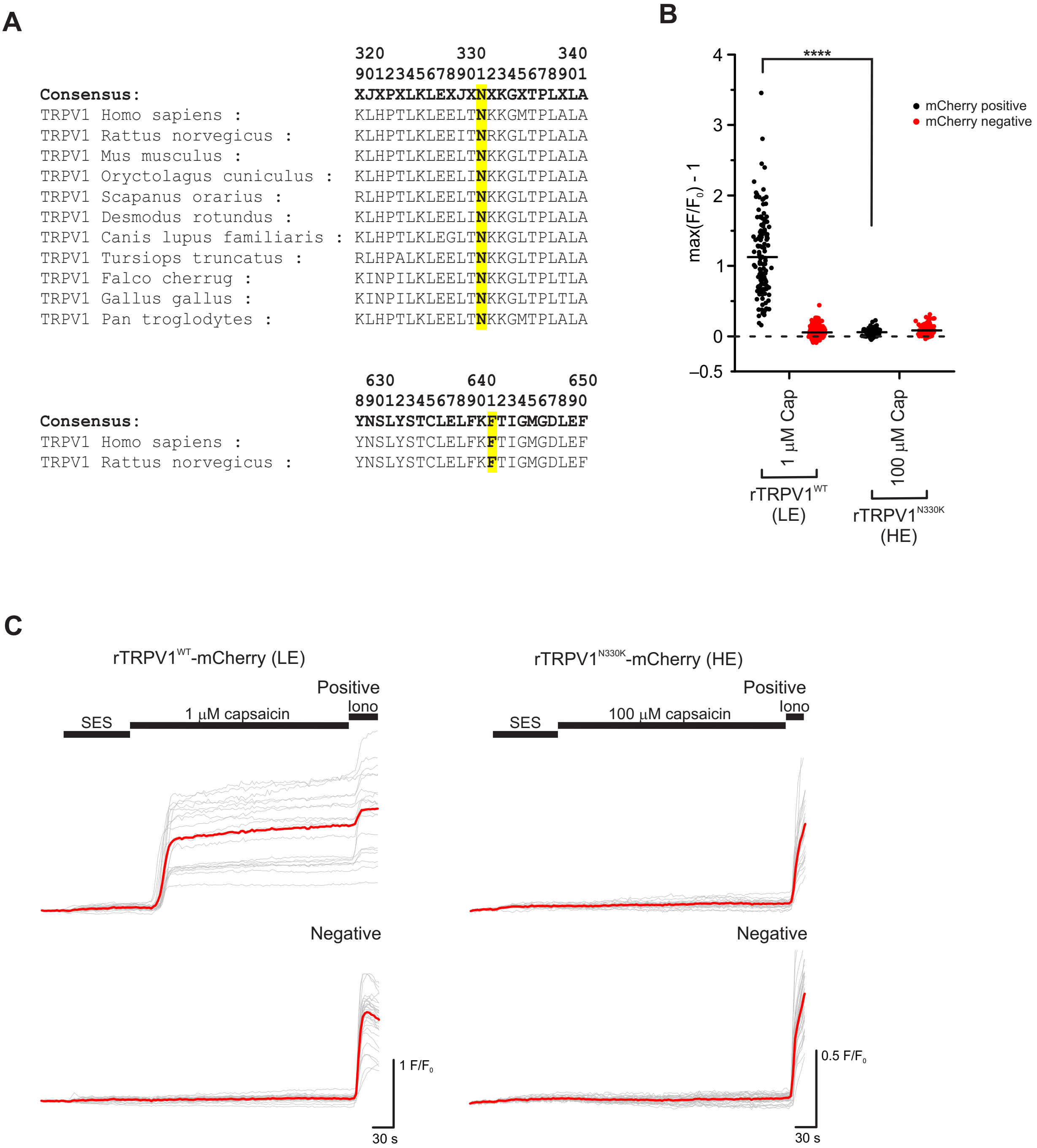
A
320 330 340
9012345678901234567890l
Consensus: XJXPXLKLEXJXNXKGXTPLXLA
TRPV1 Homo sapiens : KLHPTLKLEELTNKKGMTPLALA
TRPV1 Rattus norvegicus : KLHPTLKLEEITNRKGLTPLALA
TRPV1 Mus musculus : KLHPTLKLEELTNKKGLTPLALA
TRPV1 Oryctolagus cuniculus : KLHPTLKLEELINKKGLTPLALA
TRPV1 Scapanus orarius : RLHPTLKLEELTNKKGLTPLALA
TRPV1 Desmodus rotundus : KLHPTLKLEELINKKGLTPLALA
TRPV1 Canis lupus familiaris : KLHPTLKLEGLTNKKGLTPLALA
TRPV1 Tursiops truncatus : RLHPALKLEELTNKKGLTPLALA
TRPV1 Falco cherrug : KINPILKLEELTNKKGLTPLTLA
TRPV1 Gallus gallus : KINPILKLEELTNKKGLTPLTLA
TRPV1 Pan troglodytes : KLHPTLKLEELTNKKGMTPLALA
630 640 650
8901234567890123456789O
Consensus: YNSLYSTCLELFKFTIGMGDLEF
TRPV1 Homo sapiens : YNSLYSTCLELFKFTIGMGDLEF
TRPV1 Rattus norvegicus : YNSLYSTCLELFKFTIGMGDLEF
B
****
mCherry positive
mCherry negative
max(F/F0) - 1
4
3
2
1
0
−0.5
1 µM Cap
100 µM Cap
rTRPV1WT (LE)
rTRPV1N330K (HE)
C
rTRPV1WT-mCherry (LE)
Positive
SES
1 µM capsaicin
Iono
Negative
1 F/F0
30 s
rTRPV1N330K-mCherry (HE)
Positive
SES
100 µM capsaicin
Iono
Negative
0.5 F/F0
30 s

Supplemental Figure 9: The homologous mutation N330K renders the rTRPV1 channel inactive. (**A**) Top**:** Sequence alignment of TRPV1 from several species shows the conservation of the N331 residue. Bottom**:** Sequence alignment of hTRPV1 and rTRPV1 show the conservation of the F641 residue**.** (**B**) Maximal (max) normalized fluorescence change from baseline during application of 1 μM capsaicin (Cap): rTRPV1^{WT} LE, mCherry positive, 1 μM Cap (*N* = 7 plates, *n* = 127 cells), rTRPV1^{WT} LE, mCherry negative, 1 μM_ Cap (*N* = 7, *n* = 191) and 100 μM capsaicin: rTRPV1^{N330K} HE, mCherry positive,100 μM Cap (N = 5, *n* = 126), rTRPV1^{N330K} HE, mCherry negative, 100 μM Cap (*N* = 5, *n* = 170). ****P<0.0001 by 2-tailed Student's *t*-test. (**C**)**.** Representative traces of Fura-2 based Ca^{2+} imaging from T-REx-293 cells transiently transfected with rTRPV1^{WT}-mCherry at LE levels (left) and rTRPV1^{N330K}-mCherry at HE levels (right) in response to application of 1 μM or 100 μM capsaicin, respectively. Upper traces are from mCherry positive cells (Positive), lower traces are from mCherry negative cells (Negative). Application of the Ca^{2+} ionophore ionomycin (Iono) served as a positive control.

Table S1: Homozygous gene variants of A1 remaining after filtering and their occurrence in A2

Chr	bp (Hg19)	ref	mut	GERP	AA	Gene	MT	ExAC	gnomAD hom	Rs No.	Found also in A2
1	176668379	G	A	4.14	D-964-N	PAPPA2	D	0.0002		rs201513629	
1	179078052	T	A	5.78	T-784-S	ABL2	D	0.0031	3	rs55892721	
1	179959724	C	G	5.44	S-68-C	CEP350	D	0.0048	13	rs115186595	
1	206905941	G	T	5.27	A-361-S	MAPKAPK2	D	0.0021	5	rs55894011	
2	46597010	G	A	5.65	R-275-H	EPAS1	D	0.000009263			
2	54176305	C	T	3.04	A-120-T	PSME4	D				
2	68873308	G	A	5.21	E-119-K	PROKR1	D	0.00003374		rs140225124	Yes
2	120209611	C	T	5.33	R-299-H	SCTR	D	0.0003	1	rs376021809	
3	149459466	C	T	3.6	V-148-M	COMMD2	D	0.0032	3	rs148182261	
3	171431726	C	G	5.36	E-290-Q	PLD1	D	0.0017		rs142070781	
16	67273254	A	G	4.85	I-102-T	FHOD1	D	0.0012	2	rs138319092	
16	69748928	C	G	6.03	R-119-P	NQO1	D	0.0041	17	rs11555215	
16	69760341	A	G	3.56	M-1-T	NQO1	D	0.0042	20	rs114238154	
17	3493152	G	C	2.61	N-331-K	TRPV1	D	0.000008858			Yes
17	7557394	A	T	5.23	Q-124-L	ATP1B2	D	0.003	13	rs34745087	
20	43533771	C	T	5.87	T-196-M	YWHAB	D	0.0002		rs146766895	

Supplemental Table 1: Homozygous gene variants of A1 remaining after filtering and their occurrence in A2.

Summary of homozygous gene variants remaining after filtering in A1. **Chr** – Chromosome; **bp (Hg19)** - location in base pairs in human genome version 19; **ref** – reference; **mut** – mutation; **GERP**- conservation score; **Gene** – Gene symbol; **MT**- MutationTaster pathogenicity prediction (D: Disease causing – probably deleterious, N: polymorphism – probably harmless), **ExAC** - Exome Aggregation Consortium; **gnomAD hom** – number of homozygotes among the more than 125,000 exomes sequences in the Genome Aggregation Database (gnomAD); **Rs No.** - Reference SNP cluster ID.

Table S2: Anamnesis, Past medical History and Hematological examination

Index	A1	A2*	E13	E14	E15	E16	E17	E18	E19	E20	E21
TRPV1 Genotype	N331K/N331K	N331K/N331K	+/N331K	+/N331K	+/N331K	+/N331K	+/N331K	+/N331K	+/N331K	+/N331K	+/N331K
Year of Birth	2008	2018	1975	1978	1973	1985	2004	2004	2010	2008	2014
Gender	Male	Female	Male	Female	Male	Female	Male	Male	Male	Male	Female
Age of the participants during data collection	3-11	1-3	43-44	41	46	34	12-13	12-13	9	11	8

* - according to parents report and impressions

History	A1	A2	E13	E14	E15	E16	E17	E18	E19	E20	E21
Capsaicin Sensitivity	**N**	**N**	Y	Y	Y	Y	Y	Y	Y	Y	Y
Congenital Onset (insensitivity to "hot" pepper)	**Y**	**Y**	N	N	N	N	N	N	N	N	N
Cognitive Development	Normal	Normal	Normal	Normal	Normal	Normal	Normal	Normal	Normal	Normal	Normal
Affected Sibling	N	N	N	N	N	N	Y	Y	Y	Y	Y
Pain Injuries (Fractures, see below)	Y	N/A	Y	N/A	Y	N/A	N/A	N/A	N/A	N/A	N/A
Pain Injuries (Burns, see below)	Y	N/A	Y	Y	Y	Y	Y	Y	Y	Y	Y
Somatic and Visceral Pain (see below)	Y	Y*	Y	Y	Y	Y	Y	Y	Y	Y	Y
Inflammatory Pain and itch (see below)	Y	Y*	Y	Y	Y	Y	Y	Y	Y	Y	Y
Urinary (see below)	Normal	Normal	Normal	Normal	Normal	Normal	Normal	Normal	Normal	Normal	Normal
Bedside Temperature	Normal	Normal	Normal	Normal	Normal	Normal	Normal	Normal	Normal	Normal	Normal

Examination	A1	A2	E13	E14	E15	E16	E17	E18	E19	E20	E21
Autonomic Function (Sweating, Bowel Function, Blood Pressure)	**Abnormal (Extensive sweating)**[a]	**Abnormal (Extensive sweating) was reported at 1 year of age. This condition has improved with age (Currently Normal- mild sweating)**[a]	Normal	Normal	Normal	Normal	Normal	Normal	Normal	Normal	Normal
Intact Corneal Reflex	Y	Y	Y	Y	Y	Y	Y	Y	Y	Y	Y
Motor Examination	Normal	Normal	Normal	Normal	Normal	Normal	Normal	Normal	Normal	Normal	Normal
Deep tendon reflexes	Normal	Normal	Normal	Normal	Normal	Normal	Normal	Normal	Normal	Normal	N/A
Proprioception	Normal	Normal	Normal	Normal	Normal	Normal	Normal	Normal	Normal	Normal	Normal
Touch Ability (Vibration, Mechanical Detection)	Y	Y	Y	Y	Y	Y	Y	Y	Y	Y	Y
Hearing Ability	Y	Y	Y	Y	Y	Y	Y	Y	Y	Y	Y
Smell Ability	Y	Y	Y	Y	Y	Y	Y	Y	Y	Y	Y
Taste Ability (sweet, salt, sour, bitter, pungent compounds not included)	Y	Y	Y	Y	Y	Y	Y	Y	Y	Y	Y
Blood Sugar Levels	Normal and Normal HbA1C levels	N/A	N/A	N/A	N/A	N/A	N/A	N/A	N/A	N/A	N/A
Immunology	Normal (see table)	N/A	N/A	N/A	N/A	N/A	Normal (see table)	N/A	N/A	N/A	N/A
Small Fibers (PGP9.5 positive)	Y	N/A	N/A	N/A	N/A	N/A	N/A	N/A	N/A	N/A	N/A
Body temperature, Blood Pressure, O_2 saturation	36.6 °C[b], 130/69, O_2 = 96%	37.0 °C[c], N/A, N/A	36.5 °C, N/A, N/A	36.5 °C, N/A, N/A	N/A, N/A, N/A	N/A, N/A, N/A	N/A, N/A, N/A	N/A, N/A, N/A	N/A, N/A, N/A	N/A, N/A, N/A	36.9°C, N/A, N/A

[a] – For more in-depth questions see "Thermoregulation"
[b] – Mean of 5 measurement, which were made throughout the day time. All measurements were within normal body temperature values.
[c] – Mean of 3 measurement, which were made throughout the day time. All measurements were within normal body temperature values.

Investigation	A1	A2	E13	E14	E15	E16	E17	E18	E19	E20	E21
Histamine Flare, Itch, Pain	Y, Y, Y	N/A	N/A	N/A	N/A	N/A	N/A	N/A	N/A	N/A	N/A
AITC (mustard Oil) Flare, Itch, Pain	**Y, Y, Y (Demonstrated relatively extensive flare and pain response)**	N/A	Y, Y, Y	Y, Y, Y	Y, Y, Y	Y, Y, Y	Y, Y, Y	Y, Y, Y	Y, Y, Y	Y, Y, Y	Y, Y, Y
Capsaicin Flare, Itch, Pain	**N, N, N**	**N, N, N**	Y, Y, Y	Y, Y, Y	Y, Y, Y	Y, Y, Y	Y, Y, Y	Y, Y, Y	Y, Y, Y	Y, Y, Y	Y, Y, Y
Heat-pain behavior	**Relatively low sensitivity**	N/A	Normal	Normal	Normal	Normal	Normal	Normal	Normal	Normal	Normal
Cold-pain behavior	**Relatively high sensitivity**	N/A	Normal	Normal	Normal	Normal	Normal	Normal	Normal	Normal	Normal

QST	A1	A2	E13	E14	E15	E16	E17	E18	E19	E20	E21
Warm detection threshold	Normal	N/A	Normal	Normal	Normal	Normal	Normal	Normal	Normal	Normal	Normal
Cold detection threshold	Normal	N/A	Normal	Normal	Normal	Normal	Normal	Normal	Normal	Normal	Normal
Cold-pain threshold	**Abnormal**	N/A	Normal	Normal	Normal	Normal	Normal	Normal	Normal	Normal	Normal
Heat-pain threshold	**Abnormal**	N/A	Normal	Normal	Normal	Normal	Normal	Normal	Normal	Normal	Normal
Mechanical detection threshold	Normal	N/A	Normal	Normal	Normal	Normal	Normal	Normal	Normal	Normal	Normal
Mechanical-pain threshold	Normal	N/A	Normal	Normal	Normal	Normal	Normal	Normal	Normal	Normal	Normal
Cold tolerance (thermode)	**Abnormal**	N/A	Normal	Normal	Normal	Normal	Normal	Normal	Normal	Normal	Normal
Cold tolerance (cold pressor)	Normal	N/A	Normal	Normal	Normal	Normal	Normal	Normal	Normal	Normal	Normal
Heat tolerance	**Abnormal**	N/A	Normal	Normal	Normal	Normal	Normal	Normal	Normal	Normal	Normal
QST following topical capsaicin application	**Abnormal**	N/A	Normal	Normal	Normal	Normal	Normal	Normal	Normal	Normal	Normal
QST following topical AITC application	Normal	N/A	Normal	Normal	Normal	Normal	Normal	Normal	Normal	Normal	Normal

**Somatic and Visceral Pain:**											
**Fractures:**	**A1**	**A2**	**E13**	**E14**	**E15**	**E16**	**E17**	**E18**	**E19**	**E20**	**E21**
Did you get bone fractures? Where?	Y (hand and leg)	N	Y (right hand)	N	Y (leg)	N	N	N	N	N	N
Did you feel pain	Y	N/A	Y	N/A	Y	N/A	N/A	N/A	N/A	N/A	N/A
Did the fractured area was more sensitive to touch (Mechanical Hyperalgesia)?	Y	N/A	Y	N/A	Y	N/A	N/A	N/A	N/A	N/A	N/A
**Burns:**											
Did you get burnt?	Y	N	Y	Y	N	Y	Y	Y	Y	Y	Y
Do you get burnt frequently?	N	N	N	N	N	N	N	N	N	N	N
Did you feel pain at the burnt area?	Y	N/A	Y	Y	N/A	Y	Y	Y	Y	Y	Y
Did the burnt area was more sensitive to touch and hot water (Hyperalgesia)?	Y	N/A	Y	Y	N/A	Y	Y	Y	Y	Y	Y
**General**											
Did you feel pain inside your body (visceral pain)	Y	Y*	Y	Y	Y	Y	Y	Y	Y	Y	Y
Did you have heartburns?	Y	N/A	Y	Y	Y	Y	Y (due to excessive consumption of soft drinks)	Y	N/A	Y	N/A
Did you have abdominal pressure?	N	Y*	N	N	Y	N	Y	Y (high frequency)	Y	N	N
Did you feel stomach pain?	Y	Y*	Y	Y	Y	Y	Y	Y	Y	Y	Y
Did you have ulcers?	Y	N	N	N	N	N	N	N	N	N	N
Did you have hemorrhoids?	N	N	N	N	N	N	N	N	N	N	N
Did you have muscle pain?	Y	N/A	Y	Y	N	Y	N	N	N	Y	N
Did you have joint pain (General, fingers, thigh, knees, ankle)?	Y (ankle)	N/A	Y (knee)	Y (fingers, thigh and knee)	Y	N	N	N	N	N	N
Did you have a headache?	Y	N/A	Y	Y	Y	Y	N	N	Y	Y	Y
Did you have migraine?	N	N/A	N	N	Y	N	N	N	N	N	N
Did you feel tooth pain?	Y	N/A	Y	Y	Y	Y	Y	Y	Y	Y	Y
Did you feel tooth pain when drinking cold beverages?	Y	N/A	Y	Y	N	Y	Y	Y	Y	Y	Y
Did you have urinary infection?	Y (at least once[d])	N	N	Y (sometimes)	N	Y (once)	Y (twice)	Y (3 times)	Y (twice)	N	Y
Did you feel pain while urinating?	N (even during urinary infection[d])	N	N	Y (sometimes)	N	Y (during urinary infection)	Y (during urinary infection)	Y (during urinary infection)	Y (during urinary infection)	N	Y (during urinary infection)

[d] – The symptoms were (according to the parent report): dark and red urine color, high fever and pain at the flank. The family physician diagnosed it as urinary infection and prescribed antibiotics (ceftriaxone). No urinary culture was made. The treatment with ceftriaxone was effective. No pain while urinating was reported during the period of the disease.

Inflammatory Pain and itch:	A1	A2	E13	E14	E15	E16	E17	E18	E19	E20	E21
Did you feel pain when wounded?	Y	Y*	Y	Y	Y	Y	Y	Y	Y	Y	Y
Does the wounded area become more sensitive to touch?	Y	Y*	Y	Y	Y	Y	Y	Y	Y	Y	Y
Does the wounded area become more sensitive to hot water?	Y	Y*	Y	Y	Y	Y	Y	Y	Y	Y	Y
Did you have osteoarthritis? Did you feel pain?	N	N	N	N	N	N	N	N	N	N	N
Did you have strep throat? Did you feel pain?	Y, Y	Y, Y*	Y, Y	Y, Y	Y, Y	Y, Y	Y, Y, High frequency	Y, Y, High frequency	Y, Y	Y, Y	Y, Y
Did you have eye infection? Did you feel pain? Did it itch?	Y, Y, Y	Y, Y* (pain or itch)	Y, Y, Y	Y, Y, Y	N, N, N	N, N, N	Y, Y, Y	Y, Y, Y	Y, Y, Y	N, N, N	N, N, N
Have you been stung by mosquitos or bees? Did you feel pain? Did it itch?	Y, Y, Y	Y, Y* (pain or itch)	Y, Y, Y	Y, Y, Y	Y, Y, Y	Y, Y, Y	Y, Y, Y	Y, Y, Y	Y, Y, Y	Y, Y, Y	Y, Y, Y
Do you have allergies? Did you have rashes?	N	N	Y (Penicillin)	N	N	N	N	N	N	N	N
Do you have dry skin? Does it itch? Did you have rashes?	N	N	N	N	N	N	N	N	N	N	N
Did you have lice? Did it itch?	Y, Y	N	N	N	N	N	N	N	N	N	N
Did you have chicken pox? Did it itch?	Y, Y	N	Y, Y	Y, Y	N	Y, Y	Y, Y	N	Y, Y	Y, Y	N

Urination:	A1	A2	E13	E14	E15	E16	E17	E18	E19	E20	E21
What is the frequency of micturition (per day)?	3-4	N/A	4-5	5-7	3-4	4-5	3-4	3-4	7-8	4-5	3-5
How long is the duration of micturition (long / short)?	Long	N/A	Normal	Normal	Normal	Normal	Long	Long	Long	Long	Normal
Does the micturition has decreased force of stream?	N	N/A	N	N	N	N	N	N	N	N	N
Do you have incontinence?	N	N/A	N	N	N	N	N	N	N	N	N
Do you have micturition terminal dribbling?	N	N/A	N	N	N	N	N	N	N	N	N
Do you have nocturia?	N	N/A	N	N	N	N	N	N	N	N	N
Do you have nocturnal enuresis?	Y (rarely)	N/A	N	N	N	N	N	N	N	Y	N
Did you have dysuria?	N	N	N	N	N	N	N	N	N	N	N
Did you have haematuria?	N	N	N	N	N	N	N	N	N	N	N

The Senses:											
**Hearing:**	**A1**	**A2**	**E13**	**E14**	**E15**	**E16**	**E17**	**E18**	**E19**	**E20**	**E21**
Do you hear well?	Y	Y	Y	Y	Y	Y	Y	Y	Y	Y	Y
Do you hear whisperings?	Y	Y	Y	Y	Y	Y	Y	Y	Y	Y	Y
Do you hear buzzing?	Y (rarely)	N*	N	N	N	N	Y (rarely)	N	N	N	N
**Smell:**											
Do you smell?	Y (high sensitivity)	Y	Y	Y	Y (low sensitivity)	Y	Y	Y	Y	Y	Y
Can you identify different kind of smells?	Y	N/A	Y	Y	Y	Y	Y	Y	Y	Y	Y
Are bad smells aversive?	Y	Y	Y	Y	Y	Y	Y	Y	Y	Y	Y
Are good smells attractive?	Y	Y	Y	Y	Y	Y	Y	Y	Y	Y	Y
**Taste:**											
Taste Sensitivity (sweet, salt, sour, bitter, pungent compounds not included)	Normal	N/A	Normal	Normal	Normal	Normal	Normal	Normal	Normal	Normal	Normal
Taste recognition (sweet, salt, sour, bitter, pungent compounds not included)	Y, Y, Y, Y	N/A	Y, Y, Y, Y	Y, Y, Y, Y	Y, Y, Y, Y	Y, Y, Y, Y	Y, Y, Y, Y	Y, Y, Y, Y	Y, Y, Y, Y	Y, Y, Y, Y	Y, Y, Y, Y
**Sensitivity to pungent compounds:**											
Do you feel spiciness (other than "hot" peppers)?	Y	Y*	Y	Y	Y	Y	Y	Y	Y	Y	Y
Does it burn when eating "hot" pepper?	**N**	**N**	Y	Y	Y	Y	Y	Y	Y	Y	Y
Does tears run through your eyes, when eating "hot" pepper?	**N**	**N**	Y	Y	Y	Y	Y	Y	Y	Y	Y
Can you distinguish between different kind of spiciness? i.e. garlic, mustard, cinnamon, mint, black pepper, onion, others?	**Y, extremely sensitive to garlic onion and mint**	N/A	Y	Y	Y	Y	Y	Y	Y	Y	Y
Does tears run through your eyes, when eating one of the above? Which one?	**Y, garlic and onion**	N/A	Y, onion	Y, onion	Y, onion	Y, onion	Y, onion	Y, onion	Y, onion	Y, onion, Black pepper	Y, garlic and onion
**Sight:**											
Do you see well?	Y	N/A	Y	Y	Y	Y	N	Y	N	Y	Y
Do you wear glasses?	N	N/A	N	N	N	N	N (because it is not comfortable)	N	N (because it is not comfortable)	N	N
Corneal reflex intact?	Y	Y	Y	Y	Y	Y	Y	Y	Y	Y	Y
Can you distinguish between colors?	Y	N/A	Y	Y	Y	Y	Y	Y	Y	Y	Y
Do you see well at night?	Y	N/A	Y	Y	Y	Y	N	Y	N	Y	Y
Do you suffer from frequent headaches?	N	N/A	N	N	N	N	N	N	N	N	N
Do you suffer headaches after reading?	N	N/A	N	N	N	N	N	N	N	N	N
Do you have double vision?	N	N/A	N	N	N	N	N	N	N	N	N
Do you have blind spots?	N	N/A	N	N	N	N	N	N	N	N	N
Do you see blurry?	N	N/A	N	N	N	N	Y	N	Y	N	N

**Sense of touch:**											
Do you feel light touch?	Y	Y	Y	Y	Y	Y	Y	Y	Y	Y	Y
Do you feel smooth touch?	Y	Y	Y	Y	Y	Y	Y	Y	Y	Y	Y
Do you feel coarse touch?	Y	Y	Y	Y	Y	Y	Y	Y	Y	Y	Y
Do you feel prick touch?	Y	Y	Y	Y	Y	Y	Y	Y	Y	Y	Y
Do you feel vibrations?	Y	Y	Y	Y	Y	Y	Y	Y	Y	Y	Y
Did something heavy fell on your hand or leg? Did you feel pain?	N/A	N/A	N/A	N/A	N/A	Y,Y	Y, Y	N/A	N/A	N/A	N/A
Do you feel pinching?	Y	Y	Y	Y	Y	Y	Y	Y	Y	Y	Y
Do you feel when tickled?	Y	Y	Y	Y	Y	Y	Y	Y	Y	Y	Y
**Thermosensation:**											
Do you feel heat?	Y	Y*	Y	Y	Y	Y	Y	Y	Y	Y	Y
Do you feel painful heat?	Y	Y*	Y	Y	Y	Y	Y	Y	Y	Y	Y
Does it burn?	Y	N/A	Y	Y	Y	Y	Y	Y	Y	Y	Y
Do you feel a difference between heat and painful heat?	Y	N/A	Y	Y	Y	Y	Y	Y	Y	Y	Y
Do you feel cold?	Y	Y*	Y	Y	Y	Y	Y	Y	Y	Y	Y
Do you feel painful cold?	Y	Y*	Y	Y	Y	Y	Y	Y	Y	Y	Y
Do you feel a difference between cold and painful cold?	Y	N/A	Y	Y	Y	Y	Y	Y	Y	Y	Y
Does extreme heat or cold burn?	Y	N/A	Y	Y	Y	Y	Y	Y	Y	Y	Y
At what temperature do you prefer to take a shower? (hot/cold/extreme hot/extreme cold)?	Hot	Hot	Extreme hot	Hot	Hot	Hot	Hot	Hot	Hot	Hot	Hot

**Thermoregulation:**											
Do you have the feeling that the regulation of your body temperature is disturbed?	No, his core body temperature is maintained. He feels constantly hot.	N	N/A	N/A	N/A	N/A	N/A	N/A	N/A	N/A	N
Do you experience hot flashes?	No, he feels constantly hot.	N	N/A	N/A	N/A	N/A	N/A	N/A	N/A	N/A	N
Do you experience recurrent bouts of fever?	N	N	N/A	N/A	N/A	N/A	N/A	N/A	N/A	N/A	N
Do you have difficulty adapting to changes in temperature?	N	N	N/A	N/A	N/A	N/A	N/A	N/A	N/A	N/A	N
Do you have difficulty sensing temperature differences?	No, he is able to hold extremely hot objects.	N	N/A	N/A	N/A	N/A	N/A	N/A	N/A	N/A	N
Do you feel hot when others feel comfortable or even cold (heat intolerance)?	Yes, feels constantly hot.	N	N/A	N/A	N/A	N/A	N/A	N/A	N/A	N/A	N
Do you feel cold when others feel comfortable or even hot (cold intolerance)?	N	N	N/A	N/A	N/A	N/A	N/A	N/A	N/A	N/A	N
Do you sweat a lot (summer/winter)?	**Yes (summer and winter)**	Normal-mild	Normal	Normal	Normal	Normal	Normal	Normal	Normal	Normal	Normal-mild
Is the sweat local or general all over the body?	Local	Local	N/A	N/A	N/A	N/A	N/A	N/A	N/A	N/A	Local
Where in the body do you sweat? Hands / Feet / growing / pit arm / chest / head / back / legs / all over the body.	Mostly face and pit arm	Forehead	N/A	N/A	N/A	N/A	N/A	N/A	N/A	N/A	Pit arm
Is the sweat bi-lateral (i.e both hands / both feet etc.).	Bi-lateral	Bi-lateral	N/A	N/A	N/A	N/A	N/A	N/A	N/A	N/A	Bi-lateral
How much do you sweat? (excessive, leading to wet clothes)	Excessive (wetting clothes even in cold weather)	Normal-mild	N/A	N/A	N/A	N/A	N/A	N/A	N/A	N/A	Normal
Are you sweating uniformly throughout the day?	Y	At night during the summer	N/A	N/A	N/A	N/A	N/A	N/A	N/A	N/A	Y
Do you sweat at night? Are your clothes wet when you wake up?	Yes, the linen gets wet during the night	Yes, does not wet the clothes or linen	N/A	N/A	N/A	N/A	N/A	N/A	N/A	N/A	N
Which kind of clothes do you wear in the winter?	Summer clothes (singlet)	Winter clothes (Normal)	N/A	N/A	N/A	N/A	N/A	N/A	N/A	N/A	Winter clothes (Normal)
Do you change cloth frequently?	Y	N	N/A	N/A	N/A	N/A	N/A	N/A	N/A	N/A	N
Which blanket do you use in the winter?	Cooling (summer) blanket	Winter blanket	Winter blanket	Winter blanket	Winter blanket	Winter blanket	Winter blanket	Winter blanket	Winter blanket	Winter blanket	Winter blanket
Which blanket do you use in the summer?	No blanket	Cooling (summer) blanket	Cooling (summer) blanket	Cooling (summer) blanket	Cooling (summer) blanket	Cooling (summer) blanket	Cooling (summer) blanket	Cooling (summer) blanket	Cooling (summer) blanket	Cooling (summer) blanket	Cooling (summer) blanket
What does exuberate sweating? Heat / exercise or stress	Not exuberated	Exercise	N/A	N/A	N/A	N/A	N/A	N/A	N/A	N/A	Exercise
At what age did the extensive sweating began?	From infancy, the condition has exacerbated with age	The extensive sweating was only	N/A	N/A	N/A	N/A	N/A	N/A	N/A	N/A	N/A

		noticed during infancy									
Does anybody in the nuclear family have the same phenomenon?	N	N	N/A	N/A	N/A	N/A	N/A	N/A	N/A	N/A	N/A
Does the sweating affect social interaction with other people?	No, he showers frequently, maintains hygiene and uses perfume.	N	N/A	N/A	N/A	N/A	N/A	N/A	N/A	N/A	N
Do you refrain from doing anything because of the sweating?	N	N	N/A	N/A	N/A	N/A	N/A	N/A	N/A	N/A	N
Does it bother you?	Yes, due to frequent changing of clothes and the frequent use of perfumes.	N	N/A	N/A	N/A	N/A	N/A	N/A	N/A	N/A	N
Usually do you feel hot or cold?	Hot	N/A	Regular	Regular	Hot	Regular	Hot, even in winter	Hot, even in winter	Regular	Regular	Regular
Do you feel hot? Where along your body?	Yes, feels like "the face is on fire and in the whole body". When touching his skin, it feels hot	N	N/A	N/A	N/A	N/A	N/A	N/A	N/A	N/A	N

**Psychology:**	**A1**	**A2**	**E13**	**E14**	**E15**	**E16**	**E17**	**E18**	**E19**	**E20**	**E21**
Do you feel fear? What do you fear?	Y, voices and darkness	Y*, Loud voices	Y, the future	Y, darkeness	N	Y, Accidents	Y, darkeness	Y, darkeness	Y, darkeness	Y, darkeness	Y, bees
Do you feel happiness? What makes you happy?	Y, food and phone	Y*, Games	Y, football	Y, family	Y, success	Y, Family	Y, games	Y, games	Y, games	Y, games	Y, travel
Do you feel sadness? What makes you sad?	Y, when he does not get what he wants	Y*, Hunger	Y, poorness	Y, poorness	Y, Anger	Y, Family, losing someone	Y, violence	Y, violence	Y, violence	Y, Scary Sound	Y

* - according to parents report and impressions

**PMH – Past Medical History**	**A1**	**A2**	**E13**	**E14**	**E15**	**E16**	**E17**	**E18**	**E19**	**E20**	**E21**
General state of health	Excellent	Excellent	Bad	Reasonable	Excellent	Excellent	Excellent	Excellent	Excellent	Excellent	Excellent
Past illnesses	N	N	Y, Chronic pneumonia	Y, Kidney stones and breast tumor	N	N	N	N	N	N	N
Surgeries:	N	N	N	Y, Removal of breast tumor and ectopic pregnancy abortion	Y, leg	Y, strabismus eye surgery and gall bladder removal	N	N	N	Y, tonsils removal	N
Current medications:	N	N	Y, Asinil	N	Y, insulin	N	N	N	N	N	N
Allergies:	N	N	Y, Penicilin	N	N	N	N	N	N	N	N
Immunization:	BCG, MMR, Measles, Hepatitis B, IPV, OPV, DPT-Hib.	BCG, MMR, Measles, Hepatitis B, IPV, OPV, DPT, Rota, Penta, PCV	N/A	N/A	N/A	N/A	BCG, MMR, Measles, Hepatitis B, IPV, OPV, DPT-Hib.	BCG, MMR, Measles, Hepatitis B, IPV, OPV, DPT-Hib.	BCG, MMR, Measles, Hepatitis B, IPV, OPV, DPT-Hib.	BCG, MMR, Measles, Hepatitis B, IPV, OPV, DPT-Hib.	N/A.
Substance abuse:	N	N	N	N	N	N	Cigarettes	Cigarettes	N	N	N
Asthma:	N	N	Y	N	N	N	N	N	N	N	N
Have you fainted?	N	N	N	Y, frequently	N	N	N	N	N	N	N
Diet:	Normal	Normal	Normal	Normal	Normal	Normal	Normal	Normal	Normal	Normal	Normal
Sleep:	23:30-7:00	22:00-8:00	24:00-6:30	24:00-6:30	2:30-6:00	23:30-8:00	23:30-7:00	23:30-7:00	21:00-7:00	22:00-8:00	22:00-6:30
Birth history:	Normal	Normal	Normal	Normal	Normal	Normal	Normal	Normal	Normal	Congenital heart defect	Normal
Growth and development	N/A	Normal	N/A	N/A	N/A	N/A	N/A	N/A	N/A	N/A	Normal

ROS – Review of systems	A1	A2	E13	E14	E15	E16	E17	E18	E19	E20	E21
Do you suffer from any disease, weight loss, night sweating, tiredness, itching, rash, fever?	**Y (Extensive sweating)**	N (Normal-mild sweating)	N	N	N	N	N	N	N	N	N
**Eye:** visual changes, headache, eye pain, double vision, scotomas (blind spots), floaters or "feeling like a curtain got pulled down" (retinal hemorrhage vs amaurosis fugax)	N	N	N	N	N	Y Strabismus, Headaches	N	N	N	N	N
**Ears, nose, mouth, and throat (ENT):** Runny nose, frequent nose bleeds (epistaxis), sinus pain, stuffy ears, ear pain, ringing in ears (tinnitus), gingival bleeding, toothache, sore throat, pain with swallowing (odynophagia)	N	N	N	N	N	N Ear infection	N	N	N	N Frequent Pharyngitis (prior to tonsils removal)	N
**Cardiovascular:** chest pain, shortness of breath, exercise intolerance, PND, orthopnoea, oedema, palpitations, faintness, loss of consciousness, claudication	Y (shortness of breath, exercise intolerance)	N	Y (shortness of breath)	faintness	N	N	Y (shortness of breath, chest pain)	Y (shortness of breath, chest pain)	Y (shortness of breath, exercise intolerance)	N	N
**Respiratory:** cough, sputum, wheeze, haemoptysis, shortness of breath, exercise intolerance, asthma	Y (shortness of breath, exercise intolerance)	N	Y (cough, sputum, wheeze, shortness of breath, exercise intolerance, asthma)	N	N	N	Y (shortness of breath	Y (shortness of breath	Y (shortness of breath, exercise intolerance)	Y (wheeze)	N
**Gastrointestinal:** abdominal pain, unintentional weight loss, difficulty swallowing (solids vs liquids), indigestion, bloating, cramping, anorexia, food avoidance, nausea/vomiting, diarrhea/constipation, inability to pass gas (obstipation), vomiting blood (haematemesis), bright red blood per rectum (BRBPR, hematochezia), foul smelling dark black tarry stools (melaena), dry heaves of the bowels (tenesmus)	N	N	N	N	N	N	N	N	N	N	N
**Genitourinary:** Urinary: Irritative vs Obstructive symptoms: Micturition – incontinence, dysuria, haematuria, nocturia, polyuria, hesitancy, terminal dribbling, decreased force of stream	Y	N	N	N	N	N	N	N	N	Y (rarely nocturnal enuresis)	N
**Musculoskeletal:** pain, misalignment, stiffness (morning vs day long; improves/worsens with activity), joint swelling, decreased range of motion, crepitus, functional deficit, arthritis	Y (pain in ankles)	N	N	N	N	N	N	N	N	Y (bone pain at the leg)	N
**Integumentary:** pruritus, rashes, stria, lesions, wounds, incisions, acanthosis nigricans, nodules, tumors, eczema, excessive dryness and/or discoloration. Breast pain, soreness, lumps, or discharge.	N	N	N	Y (breast tumor non-malignant)	N	N	N	N	N	N	N

Hematology:	A1	E17
[CD3+] % Lymphocytes (Number)	68.67 (7,289)	71.92 (7,620)
[TCRgd] % Lymphocytes (Number)	**13.03 (950)**	8.49 (647)
[CD4+] % TCRα β (Number)	64.96 (4,129)	62.13 (4,337)
[CD8+] % TCRαβ (Number)	32.61 (2,073)	35.54 (2,481)
[DNTcells] % TCRαβ (Number)	1.89 (120)	1.95 (136)
[CD19+] % Lymphocytes (Number)	14.04 (1,490)	17.37 (1,840)
[CD56+CD16+] % Lymphocytes (Number)	6.73 (714)	0.44 (47)

Supplemental Table 2: Anamnesis, Past medical History and Hematological examination.

A table summarizing medical examination, clinical tests, anamnesis, past medical history and structured medical interview of the heterozygous family members (E13-21, +/N331K) and the 2 affected individuals (A1 and A2, N331K/N331K), which covers a wide variety of health conditions (Y - yes; N - no; N/A - not available, abnormalities are highlighted in red).
